# Constitutive depletion of brain serotonin differentially affects rats’ social and cognitive abilities

**DOI:** 10.1016/j.isci.2023.105998

**Published:** 2023-01-18

**Authors:** Lucille Alonso, Polina Peeva, Sabrina Stasko, Michael Bader, Natalia Alenina, York Winter, Marion Rivalan

**Affiliations:** 1 Humboldt-Universität zu Berlin, Berlin, Germany; 2 Charité – Universitätsmedizin Berlin, Corporate Member of Freie Universität Berlin and Humboldt-Universität zu Berlin, Berlin, Germany; 3 Max Delbrück Center for Molecular Medicine in the Helmholtz Association, Berlin, Germany

**Keywords:** Biological sciences, Neuroscience, Behavioral neuroscience

## Abstract

Central serotonin appears a promising transdiagnostic marker of psychiatric disorders and a modulator of some of their key behavioral symptoms. In adult male *Tph2*^*−/−*^ rats, constitutively lacking central serotonin, we tested individual’s cognitive, social and non-social abilities and characterized group’s social organization under classical and ethological testing conditions. Using unsupervised machine learning, we identified the functions most dependent on serotonin. Although serotonin depletion did not affect cognitive performances in classical testing, in the home-cage it induced compulsive aggression and sexual behavior, hyperactive and hypervigilant stereotyped behavior, reduced self-care and exacerbated corticosterone levels. This profile recalled symptoms of impulse control and anxiety disorders. Serotonin appeared essential for behavioral adaptation to dynamic social environments. Our animal model challenges the essential role of serotonin in decision-making, flexibility, impulsivity, and risk-taking. These findings highlight the importance of studying everyday life functions within the dynamic social living environment to model complexity in animal models.

## Highlights

• Brain serotonin depletion did not impact cognitive abilities in classical procedures
• Brain serotonin depletion compromised everyday behaviors in naturalistic home-cage
• Most critical behaviors resembled symptoms of impulse control and anxiety disorders
• Multidimensional testing and naturalistic conditions offered high translational value

## Graphical abstract

**Figure:**
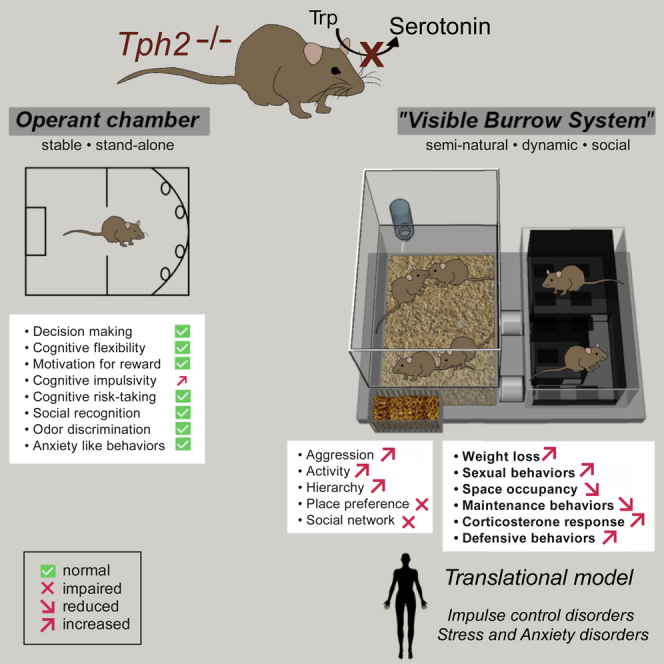

## Introduction

The complex nature of psychiatric disorders makes them some of the least understood and most incapacitating of all pathological conditions.^1^^,^^2^^,^^3^^,^^4^ A challenge for biomedical research today is to develop efficient and specific treatments that can reverse dysfunctional conditions and improve psychiatric patients’ quality of life. However, the current diagnosis of mental disorders lacks biological markers specific to given pathological conditions.^3^ Beyond the categorical classification of psychiatric disorders, the search for combinations of behavioral symptoms associated with a specific biological profile is necessary for identifying neurocognitive markers of mental disorders.^5^^,^^6^^,^^7^

The monoamine serotonin (5-hydroxytryptamine) is a neuromodulator of the central nervous system (CNS). In the CNS, its synthesis is restricted to the raphe nuclei neurons, which innervate the whole brain with a vast axonal network.^8^^,^^9^^,^^10^^,^^11^ Serotonin, through its action on numerous post- and presynaptic receptors,^12^ is essential for mood regulation and treating mood disorders (anxiety, bipolar, and depressive disorders)^10^^,^^13^ and other neuropsychiatric disorders, such as addiction,^14^^,^^15^^,^^16^ attention deficit hyperactivity disorder,^17^ suicidal behavior,^18^^,^^19^ obsessive-compulsive disorder,^20^^,^^21^ psychopathy,^22^ and other aggression-related disorders.^23^^,^^24^ At the behavioral level, serotonin is known to be critical in modulating several executive functions and aspects of social behavior. Disadvantageous decisions,^25^^,^^26^ impulsive choices and actions,^27^^,^^28^^,^^29^ inflexibility,^27^^,^^28^^,^^30^ aggression, and socially inappropriate behavior^31^^,^^32^ are characteristic impairments of affective, impulse control, or substance-related disorders.^33^^,^^34^^,^^35^^,^^36^^,^^37^^,^^38^^,^^39^^,^^40^ Similarly, such cognitive and social deficits are induced in non-clinical humans and rodents after experimental reduction of serotonin levels.^41^^,^^42^^,^^43^^,^^44^^,^^45^^,^^46^^,^^47^^,^^48^

Overall, the serotonergic system appears a promising transdiagnostic marker of apparently distinct psychiatric disorders and a common modulator of some of their key behavioral symptoms. Despite the appeal to reduce mental disorders to impairments studied in isolation, the reality is that the complexity of human mental disorders cannot be explained only in terms of their components, as their interaction plays a critical role in the emergence of the pathology.^49^^,^^50^^,^^51^^,^^52^ Using a multidimensional profiling approach,^53^ we studied the effect of brain serotonin depletion on the expression of several cognitive, social, and affective functions in the same individual. The aim of the study was to identify which of these functions were most affected by the absence of central serotonin and discuss how those key symptoms compare to mental conditions observed in humans.

Genetic modifications are among the most specific methods to target central serotonin in animals. In our study we took advantage of the recently created rats with genetic deletion of tryptophan hydroxylase 2 (TPH2),^54^ the rate-limiting enzyme for serotonin synthesis in the brain.^55^ Constitutive lack of brain serotonin in these animals^54^^,^^56^ results in delayed growth and impaired autonomic responses, which normalize at adult age. At the behavioral level, TPH2-deficient (*Tph2*^*−/−*^) rats showed increased aggression in the resident intruder paradigm.^57^ However, more subtle social and cognitive deficits remain to be characterized.

Based on previous studies where executive and social functions were individually tested after pharmacological, genetic, or dietary alteration of central serotonin, we hypothesized that the absence of serotonin would simultaneously alter the rats’ cognitive and executive functions, social abilities, activity level, and affective responses in both classical testing contexts and more dynamic home-cage environments. We used a version of a visible burrow system (VBS)^53^ to create an ethologically relevant environment and identify novel real-life markers of serotonergic function.^58^ The *Tph2*^*−/−*^ phenotype was characterized by multiple behavioral changes only detected in the dynamic social context. With unsupervised machine learning we uncovered that the most critical impairments in these animals resembled transdiagnostic symptoms of impulse control disorders.

## Results

### Central serotonin deficiency does not affect decision-making, cognitive flexibility, sensitivity to reward, motor impulsivity, social memory, and anxiety

**Figure 1:**
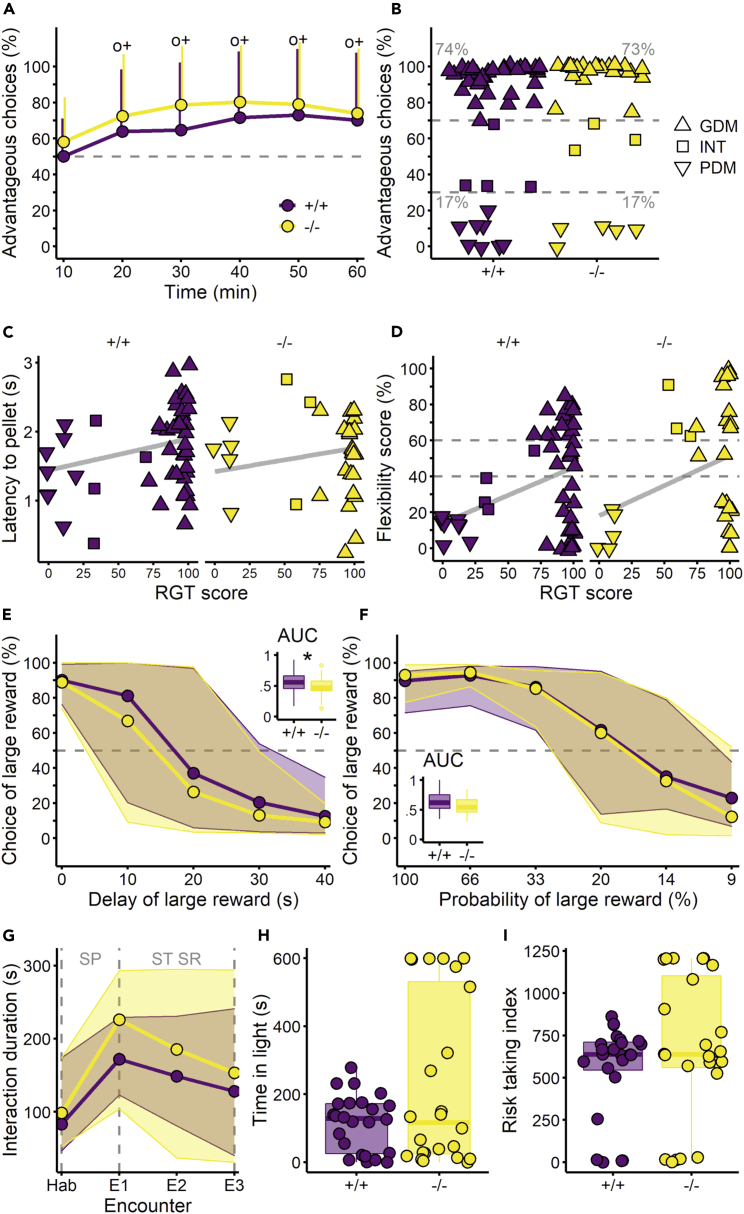
Cognitive abilities of the *Tph2*^*+/+*^ and *Tph2*^*−/−*^rats (A) Advantageous choices in the rat gambling task (RGT). Lines indicate mean +SD, one-sample t-test compared to 50% with ^o^ p value <0.05 for +/+ and ^+^ p value <0.05 for −/−. (B) Individual (mean) scores during the last 20 min of the RGT. The dashed lines at 70% and 30% of advantageous choices visually separate good decision-makers (GDMs, above 70% of advantageous choices in the last 20 min, upward triangle), intermediates (INTs, between 30% and 70% of advantageous choices in the last 20 min, square), and poor decision-makers (PDMs, below 30% of advantageous choices in the last 20 min, downward triangle). (C) Latency to collect the reward in the RGT after a choice for GDMs (upward triangle), INTs (square), and PDMs (downward triangle). Linear regression (gray line) representing the positive correlation. (D) Flexibility scores in the reversed-RGT corresponding to the preference for the new location of the preferred option in the RGT for GDMs (upward triangle), INTs (square), and PDMs (downward triangle). Linear regression (gray line) representing the positive correlation. The dashed lines at 60% and 40% visually separate flexible individuals (above 60%) from inflexible individuals (below 40%). The flexibility score is the preference for the location of the non-preferred option during the RGT. (E) Choice of the large reward option as a function of the delay in reward delivery in the delay discounting task (DDT). Lines show medians, and shaded areas show 5^th^ to 95^th^ percentiles. The dashed line indicates the 50% chance level. Inset showing the area under the curve (AUC) for the preference for the large reward, Wilcoxon rank-sum test between +/+ and −/−, ∗ p value <0.05. (F) Choice of the large reward option as a function of the probability of reward delivery in the probability discounting task (PDT). Lines show medians, and shaded areas show 5^th^ to 95^th^ percentiles. Dashed line shows 50% chance level. Inset showing the AUC for the preference for the large reward. (G) Duration of interaction in the social recognition task (SRt). Lines show the medians, and shaded areas show the 5^th^ to 95^th^ percentiles, social preference (SP), short-term social recognition (ST SR), habituation with empty cage (Hab), successive encounters with same conspecific placed in the small cage (E1–3). (H) Time in the open part of the dark-light box (DL-box). Individual data over the boxplot. (I) Risk-taking index for the DL-box test. Individual data over the boxplot. Boxplots classically represent the median, 25^th^ and 75^th^ percentiles, 1.5IQR and “outlying” points when individual data are not shown Panels A–D: +/+ n = 47, −/− n = 30, E: +/+ n = 48, −/− n = 30, F: +/+ n = 24, −/− n = 24, G: +/+ n = 30, −/− n = 30, H–I: +/+ n = 24, −/− n = 24. *Tph2*^*+/+*^ in purple and *Tph2*^*−/−*^ in yellow.

All the animals started the rat gambling task (RGT) without preference for either option (first 10 min, Figure 1A) and preferentially chose the advantageous options over the disadvantageous ones after 10 min of the test (Figure 1A, one-sample t-test, 20 min: +/+: 0.95CI [53.7, 73.9], p value = 0.008; −/−: 0.95CI [59.5, 85.2], p value <0.001 and Table S1). In both *Tph2*^*+/+*^ and *Tph2*^*−/−*^ groups, this dynamic was driven by a majority of good decision-makers (GDMs; Figures 1B and S1). Unexpectedly, both groups presented the same proportion of good (+/+: 74%; −/−: 73%), intermediate (+/+: 9%; −/−: 10%), and poor decision-makers (PDMs, +/+: 17%; −/−: 17%; Figure 1B). Regardless of their genotype but consistent with their typical decision-makers’ profile,^59^ PDMs were faster to collect rewards after a choice compared to GDMs (Figure 1C, Wilcoxon rank-sum test, *PDMs* vs. *GDMs*:*+/+*: W = 203, p value = 0.049; −/−: W = 89, p value = 0.033). PDMs were incapable of flexibility in the reversed-RGT test (Figure 1D; Wilcoxon rank-sum test, *PDMs* versus *GDMs*:*+/+*: W = 217, p value = 0.016; −/−: W = 90.5, p value = 0.028). *Tph2*^*+/+*^ and *Tph2*^*−/−*^ GDMs made either flexible choices (40% and 45%, respectively), inflexible choices (40% and 45%), or were undecided (20% and 10%, Figure 1D). GDMs and PDMs did not differ in any other tests or between genotypes (Table S2). For the remainder of the study, only genotype comparisons are presented. In the delay discounting task (DDT, Figure 1E) and probability discounting task (PDT, Figure 1F), rats’ preference for the large reward progressively decreased as the associated discounting factor (delay or uncertainty) increased. Rats of both genotypes switched preference for the (immediate) smaller reward at delay 20 s [Figure 1E, linear mixed model (lmer), *delay*: F(4, 289) = 1, p value <0.001] and at probability 20% [Figure 1F, lmer, *probability*: F(5, 202) = 173, p value <0.001]. In the DDT, *Tph2*^*−/−*^ rats presented a smaller total area under the curve (AUC) than *Tph2*^*+/+*^ animals (Figure 1E inset, Wilcoxon rank-sum test, W = 916, p value = 0.044). In the PDT, both genotypes presented similar AUC (Figure 1F inset, Wilcoxon rank-sum test, W = 373, p value = 0.081). Animals of both genotypes presented similar anticipatory and perseverative responses during the fixed-interval and extinction phases of the fixed-interval and extinction schedule of reinforcement test (FIEXT, Figure S2). Despite a similar social preference for an unfamiliar partner (E1, Figures 1G and S3) and recognition abilities (E2, E3, Figures 1G, and S3) in both groups, *Tph2*^*−/−*^ rats presented a higher interest in the social partner than the *Tph2*^*+/+*^ rats [Figure 1G, lmer, *genotype*: F(1, 40) = 8, p value = 0.006]. *Tph2*^*−/−*^ and *Tph2*^*+/+*^ rats showed similar abilities in the odor discrimination test (Figure S4). Anxiety and risk-taking levels in the dark-light box (DL-box) test were similar between genotypes, although *Tph2*^*−/−*^ rats showed high variability in responses (Figures 1H and 1I).

### Central serotonin deficiency disrupts daily activity, place preference, body weight, and corticosterone levels of group-housed rats within the VSB

**Figure 2:**
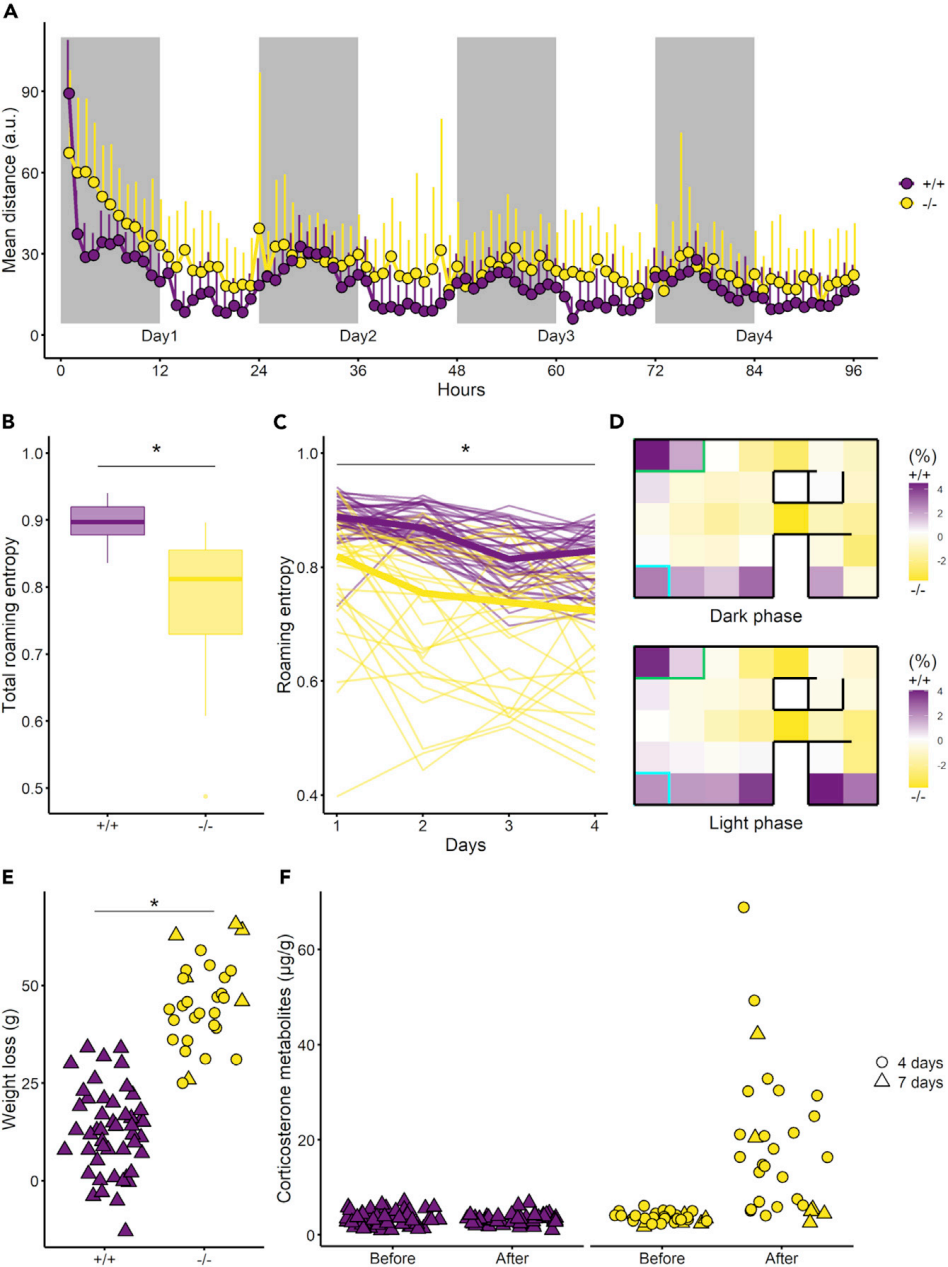
Activity, roaming entropy, and place preference of the *Tph2*^*+/+*^ and *Tph2*^*−/−*^ rats in the automated visible burrow system (VBS) (A) Activity as mean index of distance traveled in arbitrary unit per hour. Lines indicate mean +SD (B) Total roaming entropy, boxplots classically represent the median, 25^th^ and 75^th^ percentiles, 1.5IQR and “outlying” points, Wilcoxon rank-sum test between +/+ and −/−, ∗ p value <0.05. (C) Roaming entropy per day, thick lines indicate the median values, and thin lines indicate the individual values, lmer between +/+ and −/−, ∗ p value <0.05. (D) Difference in place preference (frequency of detections) in percent between +/+ and −/− over 4 days of VBS housing for dark (above) and light (below) phases. A top view of the VBS is represented and each zone corresponds to one of the 32 radio-frequency identification [RFID] detectors located beneath the VBS cage. Rectangles indicate the locations of the feeder (green) and water bottle (cyan). Positive difference (purple shade) indicates a higher place preference of the +/+ and negative difference (yellow shade) indicates a higher place preference of the −/− at each zone. (E) Weight loss in grams after the stay in the automated VBS. A 4-day stay is indicated with circles, and a 7-day stay is indicated with triangles, Wilcoxon rank-sum test between +/+ and −/−, ∗ p value <0.05. (F) Corticosterone metabolites in μg/g of feces before and after VBS housing for both genotypes. A 4-day stay is indicated with circles, and a 7-day stay is indicated with triangles; post-hoc test after lmer between before −/− and after −/− (SE= 1.4, z-value = 10.5, p value <0.001), ∗ p value <0.05. Panels A and D–F: +/+ n = 48, −/− n = 30 and B–C: +/+ n = 42, −/− n = 30. *Tph2*^*+/+*^ in purple and *Tph2*^*−/−*^ in yellow.

In the VBS, *Tph2*^*−/−*^ rats were more active than *Tph2*^*+/+*^ rats in reaction to novelty (Figure 2A, post-hoc test after lmer, day 1 – dark phase: SE = 20, z-value = 7, p value <0.001) and over days (Figure 2A, glmmMCMC, *genotype*: post mean = 8.32, credible interval [5.97, 11.03], p value <0.001). Circadian fluctuation of day/night activity was preserved in both groups (Figure 2A, glmmMCMC, *phase*: post mean = −9.14, credible interval [−9.95, −8.42], p value <0.001) although it was less pronounced for *Tph2*^*−/−*^ during light phases (glmmMCMC, *genotype x phase*: post mean = 4.13, credible interval [2.83, 5.81], pMCMC <0.001). *Tph2*^*−/−*^ rats had a lower roaming entropy (RE) index, than the *Tph2*^*+/+*^ rats overall (Figure 2B, Wilcoxon rank-sum test, W = 1183, p value <0.001) and over days (Figure 2C, lmer, *genotype*: F(1, 19) = 27, p value <0.001) indicating a more restricted use of the whole cage space than the *Tph2*^*+/+*^ rats. About place preference within the cage, *Tph2*^*−/−*^ rats were detected less often at the feeding and drinking areas and in the large chamber than the *Tph2*^*+/+*^ rats (Figure 2D, on heatmaps, the more purple the *more Tph2*^*+/+*^ rats were detected compared to *Tph2*^*−/−*^ rats). They stayed more in the covered tunnels close to the open area (burrow area) and in the center of the open area than *Tph2*^*+/+*^ rats (Figure 2D, on heatmaps, the more yellow the *less Tph2*^*+/+*^ rats were detected compared to *Tph2*^*−/−*^ rats). *Tph2*^*−/−*^ rats lost more weight during the VBS stay than *Tph2*^*+/+*^ rats (Figure 2E, Wilcoxon rank-sum test, W = 1397, p value <0.001). Only in *Tph2*^*−/−*^ rats, VBS housing largely increased the corticosterone metabolite level [Figure 2F, lmer, *genotype x time*: F(1, 94) = 69, p < 0.001].

### Central serotonin deficiency disrupts social behaviors, social networks, group organization, and hierarchy in the VBS

**Figure 3:**
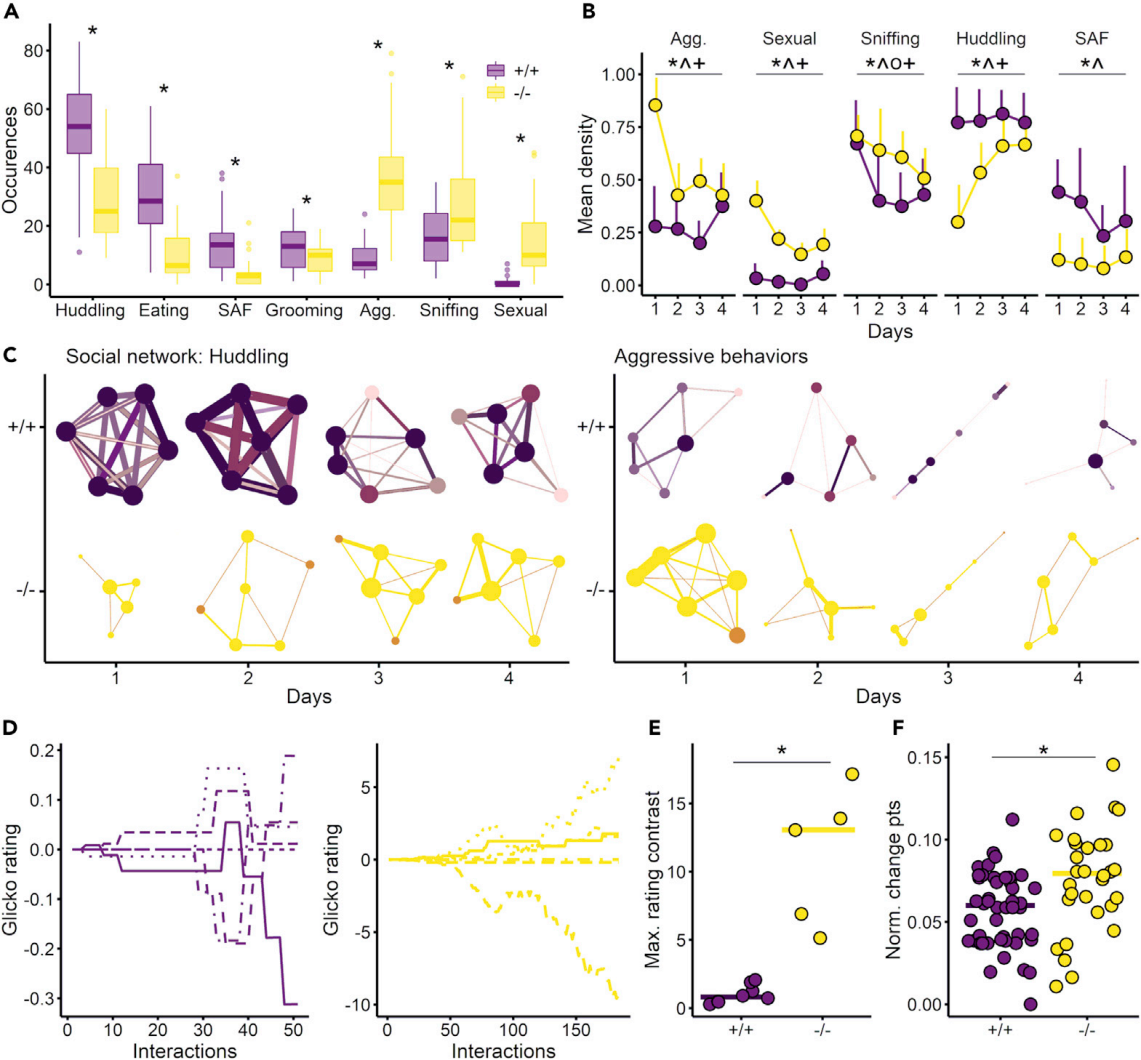
Social abilities and dominance of *Tph2*^*+/+*^ and *Tph2*^*−/−*^ in the automated VBS (A) Number of occurrences of behaviors in 4 days in the VBS for the most expressed behaviors, struggling at the feeder (SAF), general aggression including all aggressive behaviors except struggling at the feeder (Agg.), all sniffing behaviors (Sniffing), sexual behaviors including embracing and mounting behaviors (Sexual; Table 1). Boxplots classically represent the median, 25^th^ and 75^th^ percentiles, 1.5IQR and “outlying” points. Wilcoxon rank-sum test between *Tph2*^*+/+*^ and *Tph2*^*−/−*^, ∗ p value <0.05. (B) Social network density along days. Lines indicate mean +SD, lmer genotype: ∗p value <0.001, lmer genotype x day: ˆ p value <0.01, lmer day: ^o^ p value <0.001 for +/+ and ^+^ p value <0.01 for −/−. The network density is the proportion of potential connections in the network that are existing connections between rats; the development of the social network density over days can be visualized by viewing the number of edges in the networks in the panel C. (C) Representative social networks of two *Tph2*^*+/+*^ and *Tph2*^*−/−*^groups from days 1–4 for huddling (left) and aggression (right) behaviors and for illustration of data of panel B. The color intensity and thickness of the edges represent the number of behaviors exchanged (weight), and the color intensity and size of the nodes represent the number of edges received and sent out (node-degree). As in B, in this representative network of huddling, in *Tph2*^*+/+*^, the density was the highest at day 1 and remained high over days as shown by the number of edges and large node size; in *Tph2*^*−/−*^, the density was the lowest at day 1 and increased over days. In the aggression networks, in *Tph2*^*+/+*^, the density was stable and low over days; in the *Tph2*^*−/−*^ group, the density of connection strongly decreased after day 1. (D) Glicko rating representation for the six individuals of one representative *Tph2*^*+/+*^ group (left) and for the six individuals of one representative *Tph2*^*−/−*^ group (right). (E) Maximum difference in the final Glicko rating between the lowest and highest individuals (Max. rating contrast) for each group, Wilcoxon rank-sum test between +/+ and −/−, ∗ p value <0.05. (F) Individual proportion of Glicko rating change points, normalized number of change points to the total number of interaction (Norm. change pts); a change point indicates an increase or decrease in the individual rating, Wilcoxon rank-sum test between +/+ and −/−, ∗ p value <0.05. Panels A, B, and F: +/+ n = 48, −/− n = 30, panel E: +/+ n = 8 groups, −/− n = 5 groups and panels C and D representative groups of each genotype. *Tph2*^*+/+*^ in purple and *Tph2*^*−/−*^ in yellow.

Overall, *Tph2*^*−/−*^ animals showed less huddling, eating, struggling at the feeder, and grooming behaviors than *Tph2*^*+/+*^ animals and more general aggression, exploratory (sniffing), and sexual behaviors (Figure 3A, Wilcoxon rank-sum test, huddling: W = 1240.5, p value <0.001; eating: W = 1267, p value <0.001; struggling at feeder: W = 1227.5, p value <0.001; grooming: W = 914.5, p value = 0.0459; general aggression: W = 29, p value <0.001; sniffing: W = 429, p value = 0.0028; sexual behaviors: W = 67, p value <0.001, and all behaviors are presented in Figure S5 and defined in Table 1). On day 1, for aggression and sexual behavior, *Tph2*^*−/−*^ networks were more dense, with most pairs of rats displaying these behaviors, whereas fewer pairs connected for huddling and struggling at the feeder compared to *Tph2*^*+/+*^ networks [Figure 3B, lmer, *genotype*, general aggression: F(1, 43) = 40.9, p value <0.001; sexual behavior: F(1, 44) = 167, p value <0.001; huddling: F(1, 43) = 32.5, p value <0.001; struggling at feeder: F(1, 43) = 15.2, p value <0.001; and Table S3]. On the following days and by day 4, the *Tph2*^*−/−*^ network densities for huddling (Figure 3C-left representative network), sniffing, and general aggression (Figure 3C-right representative network) normalized to the level of the *Tph2*^*+/+*^ networks (Figure 3B); network densities for sexual behaviors always remained higher for *Tph2*^*−/−*^[Figure 3B; lmer, *genotype x day*: F(3,38) = 11, p value <0.001] and for struggling at the feeder remained stable for both genotypes [Figure 3B; lmer, *day*, +/+: F(3,23) = 2, p value = 0.13; −/−: F(3,14) = 0.2, p value = 0.89]. The average path length (mean number of steps between any pair of the network) indicated similar results to density, and the out-degree centralization (distribution of out-interactions) was low for all networks (median at 0.20, Figure S6). In both genotypes, individual hierarchical ranks emerged progressively (Figure 3D). The rats’ final Glicko ratings were broadly distributed below and above the initial rating score (Figure 3D), with one dominant individual identified in each group (except for one *Tph2*^*−/−*^ group with two dominant individuals, Figure S7). The two hierarchical scales, the non-aggression Blanchard dominance and the Glicko rating scores, correlated positively in *Tph2*^*+/+*^ (r = 0.30, p value = 0.0405) and negatively in *Tph2*^*−/−*^ (r = −0.45, p value = 0.0132). Compared to *Tph2*^*+/+*^, *Tph2*^*−/−*^ dominant animals were more aggressive toward subordinates (higher rank divergence; Figure 3E, Wilcoxon rank-sum test, W = 0, p value = 0.0015) and the *Tph2*^*−/−*^ group’s hierarchy was more unstable (higher number of change points; Figure 3F, Wilcoxon rank-sum test, W = 453, p value = 0.0061). Finally, in *Tph2*^*+/+*^ rats, the higher the Glicko rating, the higher the hub centrality in the general aggression network (r = 0.40, p value = 0.0051). This correlation was not found in *Tph2*^*−/−*^ rats (r = 0.04, p value = 0.8543), indicating that the dominant’s aggression did not influence this network.

**Table 1:** Ethogram of the behaviors scored in the VBS

Category	Behavior	Definition	Grouped category
Affiliative	Allogrooming	Gentle grooming of another rat that is not pinned on its back	
Affiliative	Attending	Orienting the head, ears, and possibly the whole body toward another rat	
Affiliative	Huddle	Lying in contact with another rat	
Affiliative	Sniffing – anogenital	Nose contact to the anogenital zone or base of tail of another rat	Sniffing
Affiliative	Sniffing – nose	Nose contact to the nose of another rat for longer than 1 s	Sniffing
Affiliative	Sniffing – body	Nose contact to the fur of another rat, sniffing it and exploring the other animal	Sniffing
Aggressive	Struggling at feeder	Rats pushing each other to obtain the place at the feeder	
Aggressive	Aggressive grooming	Vigorous grooming of another rat while pinning it	General aggression
Aggressive	Attack: Attack bite, jump, and lateral attack	Sudden bite toward neck and back of another rat. Sudden jump toward another rat. Arched-back posture oriented toward another rat, often including shoving and piloerection	General aggression
Aggressive	Following	Rat runs after another one	General aggression
Aggressive	Fight	Rough-and-tumble of two animals	General aggression
Aggressive	Mutual upright posture	Both rats standing in front of each other with vertical movements of the forepaw	General aggression
Aggressive	Pinning	Being above another rat usually lying on its back and holding it with the forepaw	General aggression
Aggressive	Struggle in tunnels	Rats pushing each other to pass in the tunnel, struggling with the paws	General aggression
Sexual	Mounting	Rat encircles the back, hips, or waist of another rat with its forelimb and shakes its hips	Sexual
Sexual	Embracing	Rat encircles the back, hips, or waist of another rat with its forelimb without shaking its own hips	Sexual
Defensive	Flight	Rapid movement away from another rat	
Defensive	Freezing	Being immobile or maintaining a specific posture (crouching)	
Defensive	Lateral defense	Exposing the flank to another rat	
Defensive	Supine posture	Lying on the back (exposure of the belly) because of another rat	
Defensive	Upright defense	Exposing the belly to another rat in a half-erect posture	
Maintenance	Drinking	Drinking water	
Maintenance	Eating	Eating food	
Maintenance	Grooming	Self-grooming: a rat is cleaning itself with rapid little nibbles	

### Central serotonin deficiency differentially impacts cognitive abilities and group-housed behaviors

**Figure 4:**
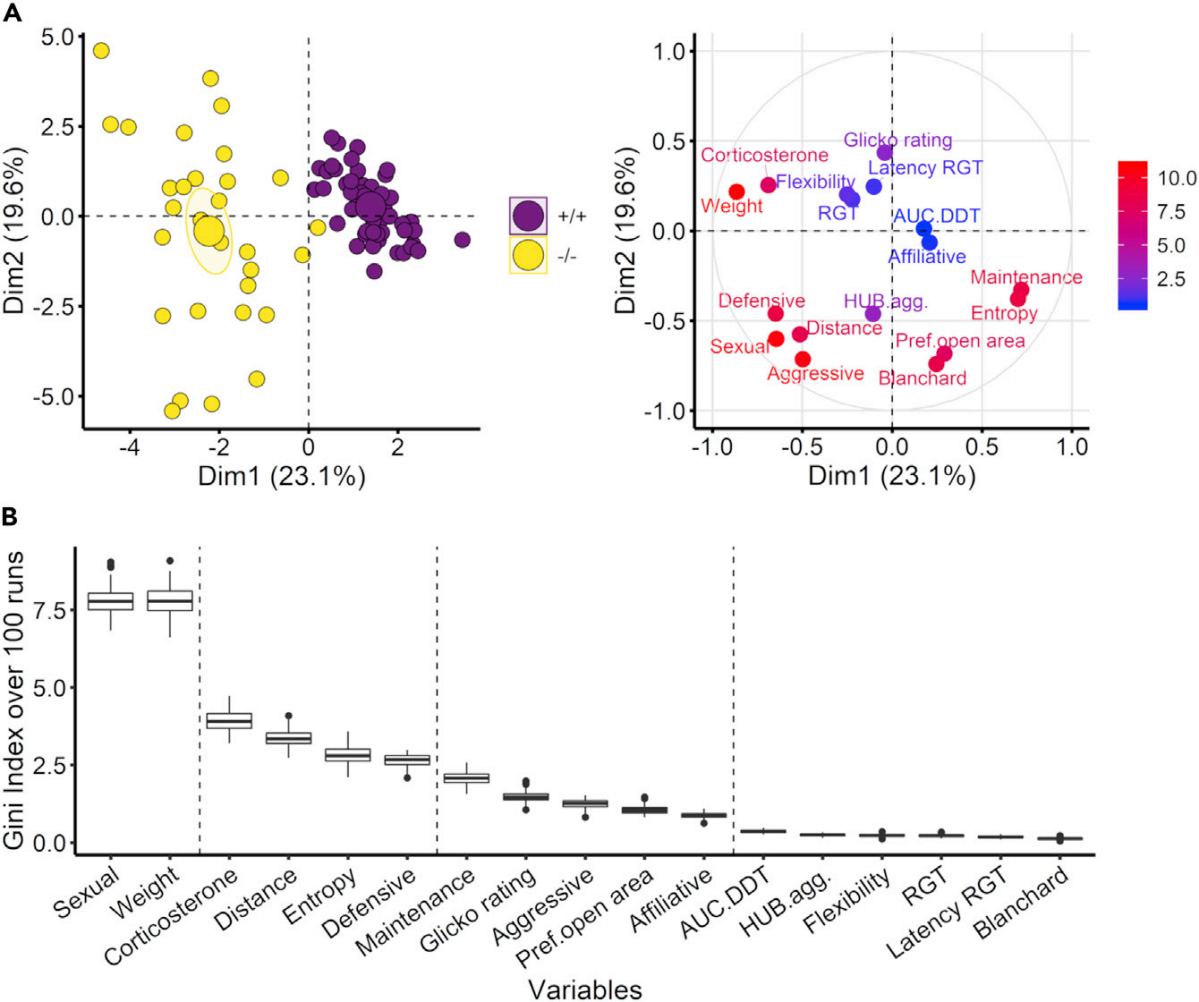
Principal component analysis (PCA) and random forest (RF) classification (A-left) Separation of the genotypes along dimension 1 but not along dimension 2 of the principal component analysis, *Tph2*^*+/+*^ in purple and *Tph2*^*−/−*^ in yellow; large symbols show group centroids and ellipses show the 0.95 confidence interval. (A-right) Contribution of the variables to dimensions 1 and 2 of the principal component analysis; higher contribution with warmer color (red, points with higher coordinates values closer to the circle) and lower contribution with colder color (blue, points with lower coordinates values closer to the center). (B) Gini index of the RF classification over 100 runs indicating the importance of the variable for the genotype dissimilarity. Boxplots classically represent the median, 25^th^ and 75^th^ percentiles, 1.5IQR and “outlying” points. The dashed line indicates the groups of variables resulting from the k-means clustering of the Gini indexes over 100 runs. Total occurrences of sexual behaviors (Sexual), percentage of weight variation (Weight), percentage of corticosterone metabolite variation (Corticosterone), total distance traveled (Distance), total roaming entropy (Entropy), total occurrences of defensive behaviors (Defensive), total occurrences of maintenance behaviors (Maintenance; drinking, eating, grooming), total occurrences of aggressive behaviors (Aggressive), total preference for the open area (Pref.open area), total occurrences of affiliative behaviors (Affiliative), area under the curve in the delay discounting task (AUC.DDT), hub centrality in aggression network (HUB.agg), flexibility score in reversed-RGT (Flexibility), preference in last 20 min of rat gambling task (RGT), latency to collect pellet in RGT (Latency RGT), Blanchard dominance score (Blanchard). Panels A–B: +/+ n = 48, −/− n = 30.

Among all measured behaviors, those most impacted by the lack of brain serotonin were identified using a random forest (RF) classifier (with an average accuracy of 98.5%, SD = 0.54, Table S4) and confirmed by a Principal component analysis (PCA, Table S5). The PCA revealed a clear separation of the genotypes along its first dimension (Figure 4A-left). The variables contributing the most to dimension 1 were also those discriminating the best between genotypes using the RF classifier (Figures 4B, Tables S6 and S7). Dimension 1 was mainly loaded by weight loss, maintenance (drinking, eating, grooming) behavior, RE, corticosterone variation, and defensive and sexual behaviors (Figure 4A-right). From the RF, the other relevant variables comprised total distance traveled, Glicko rating score, affiliative (allogrooming, attending, huddling, sniffing) and aggressive (struggling at the feeder and general aggression; Table 1) behaviors, and the presence in the VBS open area (Figure 4B). None of the cognitive variables predicted the animals’ genotypes (Figure 4A-right and B).

## Discussion

In this multidimensional study, we used classical and ethological approaches of testing to evaluate the effects of brain serotonin deficiency on the expression of cognitive, social, and affective functions in different contexts and in the same animals. With unsupervised statistics, we identified which functions were primarily affected by the absence of brain serotonin. Surprisingly, no function evaluated in the classical testing appeared altered by its absence. However, in the day-to-day context of the home-cage, the absence of brain serotonin most strikingly affected the animals’ sexual, maintenance (eating, drinking, grooming), and defensive behaviors, levels of home-cage RE, weight, and corticosterone. These discriminative markers of serotonin function, consistent with the constellation of other behavioral impairments observed in *Tph2*^*−/−*^ rats, are reminiscent of common symptoms found in human impulse control disorders (ICD; e.g. disruptive, impulse control, and conduct disorders, compulsive sexual behavior disorder, and behavioral addictions) and stress and anxiety disorders (e.g. obsessive-compulsive, post-traumatic stress, and generalized anxiety disorders), which also share a high comorbidity level with ICDs (Table S8).^60^^,^^61^^,^^62^^,^^63^^,^^64^^,^^65^

Under the complex and experimenter-free conditions of their home-cage, *Tph2*^*−/−*^ rats showed increased corticosterone levels, exacerbated repetitive aggression, and exploratory (sniffing) and sexual behaviors while neglecting affiliative (huddling), self-caring (grooming), and self-sustaining (feeding, poor maintenance of body weight) essential behaviors. Although the dynamics of interactions eventually normalized for aggressive, exploratory, and affiliative behaviors, it did not for sexual behaviors. In clinical settings, cortisol disturbances, uncontrolled repetitive violent or sexual outbursts with poor consequences for others (harm) and self (neglect of health and personal care) are characteristic of disruptive, impulse control, and conduct disorders^66^^,^^67^^,^^68^^,^^69^^,^^70^ and compulsive sexual behavior disorder.^71^ At the group level, *Tph2*^*−/−*^ dominance was emphasized by increased aggression toward subordinate. This is in line with a despotic style of hierarchy which could compare, to a certain extent, to macaques’ social organizations, and in particular the expression of escalated aggression that is found inversely dependent on serotonin turnover and controlled by serotonergic gene polymorphism.^72^^,^^73^ Nonetheless, *Tph2*^*−/−*^ hierarchical ranks appeared less stable and did not reflect in the structure of aggression networks (i.e. hub centrality) as was the case in the *Tph2*^*+/+*^ groups. *Tph2*^*−/−*^ groups were disorganized overall. In line with the work of Kiser et al. (2012)^31^*Tph2*^*−/−*^ rats might present a more reactive type of aggression with persistent sexual activity and outbursts of aggression, appearing devoid of long-term goals (e.g. reproduction, secure food resource, hierarchical structure) and of specificity (e.g. occurred between random conspecifics). In addition, *Tph2*^*−/−*^ rats expressed a hypervigilant defensive profile with higher day/night activity and smaller territories, ignoring food sources but favoring hiding and escaping options. Concerning the physiological changes, possible explanations could be that the downstream glucocorticoid receptor pathway’s disruption by serotonin depletion may have maintained elevated corticosterone levels in *Tph2*^*−/−*^ rats,^74^^,^^75^ and weight loss may have resulted from social stress-inducing feeding pattern modifications.^76^^,^^77^ Finally, the rich phenotype of the *Tph2*^*−/−*^ rats within the VBS confirmed the potential of this line to model transdiagnostic features of human disorders and revealed behavioral dysfunctions at the group level and the essential role of serotonin in modulating social and non-social daily life behaviors.

However, outside the home cage, the same animals had normal scores under the controlled conditions of cognitive testing. *Tph2*^*−/−*^ rats solved complex and risky decision-making tasks. They showed normal cognitive flexibility, typical sensitivity to reward, satisfactory motor control, good social recognition and odor discrimination abilities, and normal levels of anxiety and risk-taking. Only in the DDT, they appeared more sensitive to the discounting effect of the delay on their preference for the larger reward. Such preserved cognitive performance in the absence of brain serotonin was highly unexpected, as it contrasted with the dominant literature indicating an essential role of serotonin in modulating these higher-order functions using the same classical tests.,^30^^,^^42^^,^^78^^,^^79^^,^^80^^,^^81^^,^^82^^,^^83^^,^^84^^,^^85^^,^^86^^,^^87^ although see^88^^,^^89^^,^^90^^,^^91^^,^^92^^,^^93^^,^^94^^,^^95^^,^^96^ However, before these results might indicate a more limited role for serotonin in modulating executive functions (decision-making, impulsivity, flexibility, social recognition), it is necessary to consider other potential explanations.

The lack of cognitive impairments could be because of the specific animal model we used. Knockout models specifically target one gene.^97^ Compared to pharmacological models, they prevent potential off-target effects associated with compound specificity, dosage, and application route. In a previous study, we confirmed normal cognitive and social abilities in *Tph2*^+/+^ Dark Agouti rats,^53^ excluding the risk of a flooring effect in *Tph2*^*−/−*^ rats. However, a limitation of constitutive knockout models is their propensity to develop unexpected compensatory mechanisms, which might neutralize the genetic perturbation and result in a lack of phenotype.^98^ Following this hypothesis, TPH2-deficiency in mice and rats led to an increase in brain-derived neurotrophic factor (BDNF) levels in the hippocampus and prefrontal cortex^99^^,^^100^^,^^101^ and serotonergic hyperinnervation.^101^

Studies in *Tph2*^*−/−*^ mice showed that “serotonergic neurons” were morphologically conserved in these animals, despite their inability to produce serotonin.^102^^,^^103^^,^^104^ Considering the physiological co-transmissions of glutamate, dopamine, or GABA neurotransmitters by serotonergic neurons,^105^^,^^106^^,^^107^^,^^108^^,^^109^^,^^110^^,^^111^^,^^112^ activity of the “serotonergic circuitry” could have occurred in the absence of serotonin in *Tph2*^*−/−*^ rats. The hypothesis of such a compensatory scheme, counteracting the absence of brain serotonin in classical stand-alone cognitive tests, would suggest the existence of powerful biological targets for cognitive remediation, which remain to be studied.

Although it is unclear which compensatory mechanisms could have counterbalanced the absence of serotonin in classical tests, these mechanisms showed their limits under the less controlled, experimenter-free conditions of the social home-cage. In this more cognitively challenging and dynamic environment, *Tph2*^*−/−*^ rats presented altered daily life, social, and group behaviors compared to control rats. In classical tests, the cognitive demand is minimized to evaluating a few given functions, unlike natural environments where complex cognition is encouraged.^58^ Behavioral adaptation in social environments is known to be facilitated by serotonin through its influence on neural plasticity.^31^^,^^113^^,^^114^ Despite normal performances in classical cognitive tests, in the VBS, the highly dysfunctional social profile of *Tph2*^*−/−*^ rats indicates poor impulse control (e.g. sustained aggression), limited ability to adjust choices over time (e.g. sexual activity), and lack of goal-directed behavior (e.g. reduced eating and struggling at the feeder). Consistent with the context-specific role of central serotonin in modulating cognition,^113^^,^^114^ serotonin proved essential in supporting daily cognitive life in complex and social contexts.

Finally, an intriguing result concerns their social exploratory dynamic. Sniffing one another is a critical behavior in acquiring information,^115^ communicating dominance status,^116^ and pacifying interactions.^117^*Tph2*^*−/−*^ rats showed slower reduction of sniffing network density in the VBS and a higher interest in the social partner in the social recognition test. They might be slower at integrating and transmitting social cues and thus at adjusting their behavior. The lack of structure of the aggression network may indicate disrupted transmission of hierarchical information in *Tph2*^*−/−*^ groups. Thus, communication deficits may have played a significant role in maintaining aggression, hierarchical disorganization, social stress, and the uncertainty level of the VBS, potentiating the serotonin depletion effects. A deeper investigation of the communication strategy of *Tph2*^*−/−*^ rats would help understand which functions affected by serotonin depletion are responsible for these deficits.

To conclude, in this study, using adult *Tph2*^*−/−*^ rats, we showed that central serotonin was not essential for expressing cognitive abilities when tested in classical tests. However, central serotonin was a key modulator of essential naturalistic home-cage behaviors when living in undisturbed social groups. Context complexity must be integrated into experimental designs to investigate the role of the serotonergic system in the subtle modulation of different aspects of social and non-social behaviors. Only when facing the dynamic complexity and uncertainty of naturalistic conditions of choices were *Tph2*^*−/−*^ rats unable to adjust their behavior and were revealed as a promising model for studying transdiagnostic markers of ICDs and anxiety. The decision-making, flexibility, and impulsivity of the *Tph2*^*−/−*^ rats should be further studied under complex naturalistic conditions.^118^^,^^119^^,^^120^ In the complex social contexts, the unsupervised analysis of multidimensional results and analysis of network dynamics and hierarchy are essential additions to classical methods. They are necessary to expose the complexity of animals’ phenotypes and demonstrate the translational value of results.

### Limitations of the study

The primary purpose of this study was not to compare the different test environments but rather use a variety of tests to establish an extended behavioral profile of the *Tph2*^*−/−*^ rat model. Two limitations in the design of this study can be notified, as we were unable to apply blinding and randomization and those limitations should be addressed in future studies. There is also a strong need to include female subjects in future studies to examine or rule out potential differences with the present profile exclusively established in male rats.

## STAR★Methods

### Key resources table

**Table:** 

REAGENT or RESOURCE	SOURCE	IDENTIFIER
**Antibodies**		

Rabbit polyclonal anti-5α-pregnane-3β,11β,21-triol-20-one-CMO:BSA	Touma et al.^122^	Lab-code: 37e

**Deposited data**		

Data and analysis	This paper	https://doi.org/10.5281/zenodo.4912528

**Experimental models: Organisms/strains**		

Rats: Dark Agouti	Janvier Labs^123^	Cat#DA/HanRj
Rats: TPH2-ZFN	Kaplan et al.^54^	DA-Tph2em2Mcwi

**Software and algorithms**		

R 3.6.1	R Core Team	https://www.R-project.org/
R Studio 1.1.456	Posit	https://www.rstudio.com/categories/rstudio-ide/
PhenoSoft	Phenosys, Germany	https://www.phenosys.com/
CamUniversal	CrazyPixels, Germany	http://www.crazypixels.com/products/camuniversal

**Other**		

Sweet pellets 45 mg	TestDiet, USA	Cat#5TUL

### Resource availability

#### Lead contact

Further information and requests for resources should be directed to and will be fulfilled by the lead contact, Marion Rivalan (marionrivalan@gmail.com). Specific requests about TPH2-rats should be directed to corresponding author Natalia Alenina (alenina@mdc-berlin.de).

#### Materials availability

This study did not generate new unique reagents.

### Experimental model and subject details

#### Animals

Dark Agouti rats (originally from Janvier Labs, France^123^) and TPH2 rats^54^ on Dark Agouti background were bred at the Max Delbrück Center for Molecular Medicine (Berlin) and transferred to the experimental facility of the Charité between five and nine weeks of age. To generate experimental *Tph2*^*+/+*^ and *Tph2*^*−/−*^ animals, 13 *Tph2*^*+/+*^ dams were bred with *Tph2*^*+/+*^ males; and 3 and 12 dams of *Tph2*^*−/−*^ and *Tph2*^*+/−*^ genotype, respectively, were bred with *Tph2*^*−/−*^ males. One to five siblings per litter were taken from each dam. The *Tph2*^*−/−*^ pups showed a 10% mortality rate, whereas no preweaning loss was observed for *Tph2*^*+/−*^ and *Tph2*^*+/+*^ pups. Monoamine levels were controlled by HPLC: serotonin was undetectable in the brain of *Tph2*^*−/−*^ animals (data not shown), confirming previously published data.^54^^,^^56^ Genotyping of animals was performed according to the previously published protocol.^74^

In total, 48 *Tph2*^*+/+*^ and 30 *Tph2*^*−/−*^ male rats (24 born from *Tph2*^*+/−*^ and 6 born from *Tph2*^*−/−*^ dams) were used in the study. The *Tph2*^*+/+*^ group consisted of 10 Dark Agouti and 38 *Tph2*^*+/+*^ rats originating from the TPH2-breeding. Animals were housed in pairs of the same genotype in standard rat cages (EurostandardType IV, 38 cm × 59 cm) in two temperature-controlled rooms (22°C-24°C and 45%–55% humidity) with inverted 12-h light-dark cycles. We used 8 *Tph2*^*+/+*^ and 5 *Tph2*^*−/−*^ cohorts, 6 animals each. Groups of 12 animals (6 *Tph2*^*+/+*^ and 6 *Tph2*^*−/−*^) were tested either in the morning or in the afternoon (i.e. 24 animals per day) depending on the light cycle of the housing room (lights on at 20:00 in room 1 or 01:00 in room 2) in order to maximize the use of our four operant cages and minimize potential circadian effect (rats were all tested in RGT within 3h and 1h after start of dark phase).

Animals had *ad libitum* access to water throughout the experiment. They were fed *ad libitum* with standard maintenance food (V1534-000, Ssniff, Germany) except during the operant training and testing, when they were maintained at 95% of their free-feeding weight. After their daily operant testing rats were fed up to 20 g per animal depending on the amount of reward (sweet pellets) they received in the operant chamber and following an unpredictable schedule (one to several hours after the end of test) to avoid their anticipation of feeding. Rats were weighed every two to three days allowing for adjustment of their portion of standard food. After the VBS and before the DDT rats were given as many days as necessary to be back at 100% +/−2% of their pre-VBS bodyweight.

After staying a week undisturbed in the animal facility, animals were handled daily by the experimenters. Since*Tph2*^*−/−*^ animals were very reactive to manual handling all animals were handled using a 6 cm diameter gray polypropylene tube that was added in the cage as enrichment and used by the animals as shelter preventing fights and mounting behavior. Two weeks before the beginning of the training phase, rats were marked individually, subcutaneously in the ventral left lower quadrant with a radio-frequency identification (RFID) chip (glass transponder 3 × 13 mm, Euro I.D.) under short isoflurane anesthesia. Rats were between 8 and 14 weeksold when first trained in the operant procedures.

#### Ethical statement

All procedures followed the national regulations in accordance with the European Union Directive 2010/63/EU. The protocols were approved by the local animal care and use committee (LaGeSo Berlin) and under the supervision of the animal welfare officer of our institution.

### Method details

The study was reported in accordance with the ARRIVE Guidelines^124^ (Table S9). Numbers of animals for each test are reported in the Table S10. The number of animals was decided following *a priori* power analysis (n = 51, G∗power 3.1.2). It was reduced because of the difficulty to obtain *Tph2*^*−/−*^ animals due to their higher post-natal mortality rate. Unless stated otherwise, rats were trained and tested following established procedures described previously.^53^ The order of the tests and inter-test pauses were chosen to minimize any interference of one test on another (Figure S8). Training and testing started 1 h after the beginning of the dark phase. Animals were habituated to the experimental room conditions for 30 min before the start of the test. The order of testing of the animals was mixed and balanced in order to minimize potential confounders such as the time of the day or experimenter-related factors. A randomly generated sequence was not used for that. Blinding of the experimenter to the genotype of the animals was not possible during the conduct of experiment due to important behavioral differences at baseline. Automatic outcome assessment was used for data collection for all tests except dark light box test, social recognition task, odor discrimination test and video scoring of the visible burrow system test.

#### Operant system

Four operant cages (Imetronic, France) were used with either a curved wall equipped with one to four nose-poke holes or a straight wall equipped with one central lever, depending on the test. On the opposite wall was a food magazine connected to an outside pellet dispenser filled with 45 mg sweet pellets (5TUL, TestDiet, USA). A clear partition with a central opening in the middle of the operant cage ensured an equal distance to all nose-poke holes from this central opening for an approaching rat.

#### Rat gambling task

We used the rat gambling task (RGT) to assess complex decision-making. The operant cages were equipped with four nose-poke holes on the operant wall. The training 1 started with the four nose-pokes lit and active, a single nose-poke generated the delivery of one pellet. The selected hole remained lit until the collection of the pellet into the magazine while all the other holes were inactive. A visit to the magazine induced the reactivation and illumination of all the nose-poke holes. The training 1 continued daily until rats obtained 100 pellets in a session (30 min cut-off), then they could start the training 2. In training 2, two consecutive nose-pokes at the same hole were required to obtain one pellet and the same criterion had to be reached (100 pellets in 30 min cut-off). In training 3, two pellets were delivered after a choice (two consecutive nose-pokes) during a short session (maximum 30 pellets and 15 min cut-off). A forced training^53^ was applied to counter any side preference developed during the training procedure: if the choices for the two holes of one side were superior to 60% during the last session of training 2. During the first part of the forced-training, the two nose-poke holes on the non-preferred side were active and lit, two consecutive nose-pokes into the active holes induced the delivery of one pellet. The holes on the preferred side were inactive and not lit. After the collection of 15 pellets, the second part of the forced training started with the four holes active and lit. Two consecutive nose-pokes into holes of the preferred side induced the delivery of one pellet with a probability of 20% whereas choosing the non-preferred side induced the delivery of one pellet with a probability of 80%. The cut-off was 50 pellets or 30 min. The training procedure lasted six to ten days and the test was performed the next day.

During the test, each of the four holes was associated with an amount of reward and a possible penalty (time-out) which was unknown to the rat. Two holes on one side were rewarded by two pellets and associated with unpredictable long time-outs (222s and 444s with the probability of occurrence ½ and ¼ respectively), in the long term those options were disadvantageous. The two holes on the other side were rewarded by one pellet and associated with unpredictable short time-outs (6s and 12s with the probability of occurrence ½ and ¼ respectively), in the long term those options were advantageous. After a choice (two consecutive nose-pokes), the reward was delivered and the selected hole remained lit until a visit to the magazine or the duration of the time-out. During this time all the nose-poke holes were inactive. The test lasted 1 h (or cut-off 250 pellets). The theoretical maximum gain of the advantageous options was five times higher than the disadvantageous options at the end of the test (60 min). The percentage of advantageous choices for the last 20 min of RGT was used to identify good decision-makers (GDMs) > 70% of advantageous choices, poor decision-makers (PDMs) < 30% of advantageous choices and intermediate animals. The percentage of advantageous choices per 10 min indicated the progression of the preference over time. An index of the motivation for the reward was measured as the mean latency to visit the feeder after a choice.

#### Reversed rat gambling task

We used the reversed rat gambling task (reversed-RGT) to assess cognitive flexibility. The animals were tested in the reversed-RGT 48 h after the RGT. The same advantageous and disadvantageous options as in the RGT were used but they were switched from one side to the other. The test lasted 1 h (or cut-off 250 pellets). A flexibility score was calculated as the preference for the location (side) of the non-preferred options during the RGT. Flexible rats had >60% of such choices during the last 20 min, undecided rats had between 40% and 60% of choices, and inflexible rats had <40%. Inflexible animals are unable to adjust their behavior to follow the options previously preferred (RGT) but rather keep choosing at the same location (indifferent of the outcomes newly associated with the nose-pokes) as established before.

#### Delay discounting task

We used the delay discounting task (DDT) to assess cognitive impulsivity. The operant cages were equipped with two nose-poke holes the furthest from each other on the operant wall. One nose-poke hole (NP1) was associated with a small immediate reward (1 pellet) and a second nose-poke hole (NP5; 25 cm between the two holes) with a large (5 pellets) reward. During the training, the large reward was obtained immediately (delay 0s) after the choice (two consecutive nose-pokes). After the pellet delivery, the magazine and house lights were turned on for a 60s time-out. The session lasted 30 min (or cut-off 100 pellets). A percentage of choice of the large reward ≥70% on two following sessions with ≤15% variation (stability criterion) was required to start the test. Minimum three training sessions were done. During the test, choosing NP5 induced the delivery of the large reward after a designated delay, NP5 stayed lit during the duration of the delay. After the pellet delivery of the large reward the magazine and the house lights were turned on for a time-out of 60s minus the duration of the delay. The delay was fixed for a day and increased by 10s from 0s to 40s according to a stability criterion ≤10% variation of choice of the large reward during two consecutive sessions. The test sessions lasted 60 min (or cut-off 100 pellets). The preference for the large delayed reward was calculated as the mean percentage of NP5 choices during two stable sessions. To calculate AUC which represents the sensitivity to delay, for each individual the preference for the large delayed reward for each delays was normalized to the preference for the large delayed reward during the training and plotted against delay as a proportion of maximum delay^125^; the area under this normalized curved was then calculated.

#### Probability discounting task

We used the probability discounting task (PDT) to assess risky decision-making. The operant cages were equipped with two nose-poke holes the furthest from each other on the operant wall. This test is an adaptation of the test of Koot et al., 2012^42^ previously described in Alonso et al., 2019^53^ with the addition of a stability criterion. During the training, the large reward was always delivered after choosing NP5 (probability p = 1), which allowed the rats to develop a preference for NP5. Two consecutive nose-pokes induced the delivery of the reward after 4s, during this time the selected hole stayed lit. Then the magazine light turned on for a 15s time-out. The session lasted 25 min (or cut-off 100 pellets). A percentage of choice of the large reward ≥70% on two following sessions with ≤15% variation (stability criterion) was required to start the test. At least three training sessions were done. During the test, the probability we used were p = 0.66, 0.33, 0.20, 0.14 and 0.09. Probabilities were generated by a constant pseudo-random sequence of reward and omission. There was a non significant variation between experienced probability and theoretical probability (Figure S9). The probability was fixed for a day and increased the next day only after reaching the stability criterion of ≤10% variation of choice of the large reward during two consecutive sessions. This stability criterion ensured stability in the individual performance at a given probability. The session lasted 25 min (or cut-off 100 pellets). The percentage of preference for the large and uncertain reward was calculated for each probability as the percentage of NP5 choices during the two stable sessions. To calculate the AUC which represents the sensitivity to probabilistic uncertainty and risk taking, for each individual the preference for the large reward for each probability was normalized to the preference for the large reward during training and plotted against probabilities expressed as odds^126^ with odds = (1/P) −1; the area under this normalized curved was then calculated.

To further study impulsivity and compare the respective traits assessed in DDT and PDT, the use of an unbalanced-DDT design^127^ (with unique time-out duration) is possible to considered. Another version of the PDT offering a fully stochastic generation of reward delivery and omission is also available to mimic casino games’ settings and gamblers’ experience.^128^

#### FIEXT schedule of reinforcement test

We used the fixed-interval and extinction schedule of reinforcement test (FIEXT) to assess motor impulsivity. The operant cages were equipped with a central single nose-poke hole or a single lever. The fixed-interval (FI) consists of two phases: a fixed time interval during which choices are not rewarded, followed by a phase where a choice can be rewarded.^129^ The extinction (EXT) is a longer, fixed time interval during which no choices are rewarded. Both FI and EXT are conditions that cause frustration in the animal. A session consisted of the repetition of seven FI and one EXT of 5 min. The maximum number of pellets was 14 during a single session. FI lasted 30 s for the first four sessions, 1 min for the next four sessions, 2 min for the next three sessions and 1 min for the final four sessions. The final four sessions with a 1 min FI were the actual test. During the FI, the house light was on and the central nose-poke hole was inactive. At the end of the FI, the house light turned off and the central nose-poke was lit and became active; two consecutive nose-pokes induced the delivery of one pellet, the central nose-poke light was turned off and the tray light was lit. A visit to the tray induced the start of the next FI. After seven consecutive FI, the EXT period started, with all lights off and no consequences associated with nose poking.

When the operant cages were equipped with a lever, the scheme was similar. During the FI, the house light was on and any press on the lever had no consequence. At the end of the FI, a cue light above the lever turned on and the first press was rewarded by a pellet. The cue light above the lever stayed on until pellet collection. A visit to the tray induced the start of the next FI. After seven repetitions of the FI and pellet collection the EXT started. During EXT the house light was off and any press on the lever had no consequence.

As described earlier,^130^ the data from the first FI of the session and the first FI after the first EXT were excluded. The total number of nose pokes and mean number of nose pokes were determined for each FI and EXT period. We summed nose pokes for 10 s intervals during FI to visualize the anticipatory activity of the rats. Likewise, we summed nose pokes for 1 min intervals during EXT to visualize the perseverative activity.

#### Social recognition task

We used the Social recognition task (SRt) to assess social preference and social recognition memory. The test took place in a square open field (OF, 50 × 50 cm), a small cage was placed in one corner of the OF. To improve the setup, a foam PVC partition was placed around this intruder’s cage to avoid the test rat hiding behind the cage. The unfamiliar conspecifics were older male Wistar Han rats, accustomed to the procedure. A video camera on top of the OF recorded the experiment. Each rat was tested on two consecutive days. On the first day, the subject was placed in the OF containing the empty cage in a corner for a habituation of 15 min. Then, the unfamiliar conspecific was place in the small cage and the subject was allowed to freely explore the open field for 5 min (E1). After that the small cage with the conspecific was removed from the open field, and the subject remained alone in the open field for a break of 10 min. The encounter procedure was repeated two more times with the same conspecific (E2, E3). On the second day, the first 15 min habituation phase was followed by a fourth encounter (E4) of 5 min encounter with the same conspecific as in day 1. After this encounter, a break of 30 min took place, in which the subject remained alone in the open field. Then, the last encounter took place, but a new unfamiliar conspecific was placed in the same small cage for 5 min (Enew). The time spent in close interaction with the intruder was measured for each encounter and for the first 5 min of Habituation (Hab) when the subject smelled at the grid of the empty cage. The social preference was calculated as the ratio of the interaction time in E1 and Hab. The short-term social recognition was calculated as the ratio of the interaction time in E1 and E3. The long-term social recognition was calculated as the ratio of the interaction time in E4 and Enew.

#### Odor discrimination test

We used the odor discrimination test to assess odor discrimination ability. The test took place in a square OF (50 × 50 cm). Two plastic petri dishes filled with either spoiled (from male older Wistar Han rats) or fresh bedding were placed in two opposite corners of the OF. A video camera on top of the OF recorded the experiment. The test rat explored the OF for 5 min. The time spent in close interaction with each dish was measured and the preference for the spoiled bedding (social odor) was calculated.

#### Dark-light box test

We used a box of 45 cm × 22.5 cm x 35 cm with two compartments, one compartment made of transparent plastic was bright and one compartment made of black opaque plastic and with a lid of the same material was dark. A gate (9 cm × 10 cm) enabled the rats to pass from one compartment to the other. Room light was on and extra lamps were positioned above the box providing a high light intensity in the bright compartment >500 lux. Inside the dark box there was no appreciable illumination (i.e. 2 lux). The rat was brought into the bright compartment (with the home-cage tube) and allowed to explore the apparatus for 10 min. After the test, the apparatus was cleaned with 5% ethanol before the next rat was assessed. We recorded each tests with a video camera placed above the bright compartment. We measured the number and duration of visits to each compartment, number of risk assessments which included head poking through the door and body stretches, the latency to leave the bright compartment the first time and the duration of the first visit to the dark compartment. Risk taking index^131^ was calculated as the sum of the duration of the first visit to the dark compartment, the number of risk assessment into the light compartment and the time spent in the dark compartment, for clarity this number was subtracted to the maximum score in order to get ascending values.

#### Automated visible burrow system

We used the automated visible burrow system (VBS) to assess spontaneous social and non-social behaviors, activity, spatial occupation (see also roaming entropy), social hierarchy (see glicko rating and blanchard dominance score), social network analysis (see social network analysis) and physiological responses (see also corticosterone metabolite measurements). The automated VBS consisted of an open area connected through two transparent tunnels to a burrow system. Food and water were available at all time in the open area. The burrow system was kept in the dark throughout the test (infrared-transparent black plastic) and comprised a large and a small chamber connected by tunnels. A grid of 32 RFID detectors was placed underneath the VBS in order to automatically determine individual animal positions using the program PhenoSoft (PhenoSys, Germany). An infrared camera (IP-Camera NC-230WF HD 720p, Tri-Vision Tech, USA) mounted above the VBS recorded a 30 s video every 10 min (CamUniversal, CrazyPixels, Germany). The software PhenoSoft ColonyCage (PhenoSys, Germany) was used to superimpose colored dots (one color per animal) to the videos to allow visual identification of each individual of the group. Six rats of the same genotype were housed in the VBS for seven days in a humidity- and temperature-controlled room (temperature 23–24°C, humidity 45–50%) containing two VBS systems. The animals were visually checked every day. After the first cohort (6 *Tph2*^*+/+*^ and 6 *Tph2*^*−/−*^), the duration of the VBS housing was reduced from seven to four days^132^ for the *Tph2*^*−/−*^ animals due to noticeable weight loss. The videos of the first 4 h of the dark and light phases were scored by trained experimenters using a scan sampling method.^133^ For each rat at a time, and for each behavior expressed that was listed in Table 1,^133^^,^^134^^,^^135^^,^^136^ the experimenter reported in a behavioral ethogram 1) the type and the 2) duration of the behavior, 3) where it took place in the cage and 4) the ID of the receiver (*i.e.* the rat with which the focal rat was interacting with during the behavior). All six animals in a video were observed, one focal animal at a time. The videos were scored by three trained observers, trained together to specifically and similarly recognize the behaviors described in Table 1. The same observer scored all videos of a given group of rats. Consistency between observers was evaluated as such: for each group, one observer would randomly select 10 videos of experimental day 1 she did not yet annotate, score these videos and compare her results with the other observer’s results. If results differed, the two observers discussed discrepancies and adjusted their scorings’ strategies accordingly before further scoring. This was repeated until scorings were similar between observers. All aggressive behaviors except “struggling at feeder” were grouped under “general aggression” and sexual behaviors grouped under “sexual” (Table 1). We present the most expressed behaviors (median >5): huddling, sniffing, eating, grooming, general aggression, struggling at feeder and sexual. All scored behaviors (Table 1) are shown in the Figure S5. The body weight of the animals was measured before and after VBS housing (4 or 7 days); the difference of weight was calculated. Although wounds were rarely observed during this study, they were documented at the end of VBS housing. The activity (distance traveled) and the place preference were extracted using the software PhenoSoft analytics (PhenoSys, Germany) for the first four days of VBS housing. The time spent in the open area of the VBS was measured using the data collected from the grid of detectors.

#### Glicko rating

For each VBS group, the social ranking of the rats was defined using a Glicko rating system.^137^^,^^138^ The individual rank was dynamically updated for each individual following the outcome of each aggressive and sexual interaction during the dark phase (R package PlayerRating)^138^^,^^139^ within the group. The direction of the interaction defined the winning animal (initiator) and losing animal (receiver). We considered all types of aggressive and sexual behaviors for the first four days because both aggressive and sexual behaviors elicited defensive behaviors sometimes together with vocalizations from the receiver indicating perceived threat from the receiver. We detected the change points of the Glicko rating over time for each individuals (R package online CPD)^140^ and determined the stability of the rating. Because the total number of agonistic interactions varied between VBS groups, we calculated a normalized number of change points dividing by the group total number of interactions. For each group the divergence or maximum rating contrast was the difference between the highest and the lowest individual final ratings. Dominant animals’ ratings were higher than 1/3 of the maximum rating contrast of the group.

#### Blanchard dominance score

The Blanchard dominance score^141^ is a dominance score established in the original VBS. It originally combines three classical parameters: the number and location of wounds, the time spent in the open area and the weight loss. A wound is a visible alteration of the skin of an animal such as scratches and scabs. A wounded animal was monitored closely until complete skin healing. In our study wounds rarely occurred. Over the 78 rats tested, only nine rats presented one to 6 wounds in total (over 4 to 7 days in VBS). Because of its sporadic occurrence, the number of wounds could not be considered in the calculation of the Blanchard dominance score. For each individual within a group, time spent in open area and weight loss for the entire stay in the VBS (4 or 7 days) were ranked from 1 to 6, the average of both ranks was the Blanchard dominance score.

#### Roaming entropy

The Roaming Entropy (RE) within the VBS is the probability that an individual will be at a certain place at a given time. RE indicates the spatial dispersion of the rats within the automated VBS with high RE, indicating broader use of the cage space. RE calculation was based on the method described previously.^142^ Continuous location recordings from the RFID grid were cleaned and filtered; we selected the data from the dark phase of the first four days. We sliced the data into 1 s detections for each rat in order to weigh longer detections. We calculated the observed frequencies or probabilities, *p*_*i,j,d*_ of detection of each animal *i* at each reader *j* on a day *d*. These frequencies were then used to compute the RE for each day, following the equation of Shannon: RE_*i,d*_ = – Σ (*p*_*i,j,d*_ log *p*_*i,j,d*_)/log(k) where k is the number of detectors in the automated VBS. In the VBS, the spatial dispersion of the rats was evaluated through the total and daily RE.

#### Social network analysis

We developed the method to social network analysis to understand the qualitative aspects of the social interactions between the individuals. It allows uncovering individual and group dynamics such as information transmission or power distribution. Behavioral interactions between two individuals were organized into matrices for each category of behavior (huddling, sniffing, struggling at feeder, aggression, and sexual behavior). The matrices were weighted and directed, meaning that the number of occurrences of interactions was used and that all interactions weren’t always reciprocal in a pair of rats. We used the R package igraph^143^ to calculate the parameters and visualize the networks. We measured three global network parameters: density, average path length and out-degree centralization to understand the structure of the networks.^138^ Density is the proportion of possible ties that can exist in the network. Average path length is the mean number of steps between any pair of individuals in the network. Out-degree centralization indicates the differences of initiated connections between the individuals. We measured five individual network parameters: in- and out-degree, betweenness centrality, closeness centrality, Bonacich’s power centrality and Hub centrality, to understand the roles of individuals within networks.^138^ In- and out-degree is the number of interactions an individual receives and initiates respectively. Betweenness centrality indicates how much an individual connects two other individuals. Closeness centrality indicates how much an individual directly connects with other individuals. Bonacich’s Power Centrality defines the influence of an individual based on the connections of its neighbors, powerful individuals are connected to many individuals that themselves are less connected to others.^144^ Hub centrality also depends on the connection of an individual’s neighbors, powerful individuals (authorities) are connected to many individuals highly connected to others (hubs).^145^

#### Corticosterone metabolite measurements

One day before and immediately after VBS housing, both times at the same time of the day,the rats were housed in individual cages with food, water and clean bedding for 4 h maximum. Every 30 min, feces produced were collected in microtubes and stored at −20°C until extraction. Then, the samples were defrozen, 0.1g of feces was added to 0.9 mL of 90% methanol, agitated for 30 min and then centrifuged at 3000 rpm for 15 min. A 0.5 mL aliquot of the supernatant was added to 0.5 mL of water, this extract was stored at −20°C. Measurements of corticosterone metabolites with a 5α-3β,11β-diol structure were performed with enzyme immunoassay (EIA) using a polyclonal antibody (rabbit) against 5α-pregnane-3β,11β,21-triol-20-one (linked to carboxymethyloxim) coupled with BSA^122^ following the method of Lepschy et al.^146^ in the laboratory of Dr. Dehnhard at the Leibniz Institute of Zoo and Wildlife Research. Briefly, a double antibody technique was used in association with a peroxidase conjugate generating a signal quantitatively measurable by photometry. Corticosterone metabolite concentrations were expressed in micrograms/grams of feces.

### Quantification and statistical analysis

R (version R – 3.6.1)^147^ and R studio (version 1.1.456) were used for statistical analyses. Before comparing the genotypes, we compared the performance of Dark Agouti (n = 10) and *Tph2*^*+/+*^ from the TPH2-breeding (n = 38) animals in all the tests using the Wilcoxon rank-sum test. The results from Dark Agouti and *Tph2*^*+/+*^ from the TPH2-breeding animals were not different and these animals were grouped together to form the *Tph2*^*+/+*^ group (control group, n = 48). During the data analysis the experimenter was not blind to the genotype of the animals. We do not expect our data to follow a normal distribution; hence we used non-parametric statistical tests. We used the Wilcoxon rank-sum test to compare the two genotypes (*Tph2*^*+/+*^vs. *Tph2*^*−/−*^) against each other, the Fisher exact test to compare the number of GDMs and PDMs in *Tph2*^*+/+*^ and *Tph2*^*−/−*^ groups, the one sample t-test to compare the performance of the animals to a theoretical value in RGT and the Wilcoxon sign test (R package RVAideMemoire)^148^ to compare the performance of the animals to a theoretical value in DDT, PDT, SRt and odor discrimination test. Differences in performance between GDMs and PDMs were evaluated with the cohen’s effect size (R package effsize).^149^ Linear mixed-effect models (lmer models) can be used robustly on non-normal data.^150^ We used lmer models (R package lmerTest)^151^ to compare genotypes (or decision maker groups) over several time points and with individual and batch information as nested random effects. Post-hoc multiples comparisons were done on the linear models (R package multcomp),^152^ there the pvalues were adjusted using the Holms method for multiple comparisons. Because of their ability to model over-dispersion, we used generalized linear models with Markov chains (MCMCglmm; R package MCMCglmm)^153^ to compare the distance traveled of genotypes over light cycles and hours with individual and batch information as random effects. The fitting of the MCMCglmm models was assessed with the plots of the fixed effects and random effects. The lower deviance information criterion (DIC) was used to choose the best MCMCglmm model. We used Spearman’s correlation (R package Hmisc)^154^ to assess the link between hierarchy variables (Glicko and Blanchard scores), individual SNA centrality, roaming entropy and corticosterone level after VBS stay. For all tests, p < 0.05 was considered as statistically significant. Symbols, such as ∗ˆ^o+^, represent significant pvalue and may differentiate several comparisons on the same figure.

RF and PCA were used to identify the functions most affected by brain serotonin depletion in tests. The RF (R package randomForest)^155^ predicts the genotype of each individual based on their scores in each test and returns the importance of each variable for the classification. We used a Leave-One-Out cross-validation and ran the RF for 100 runs. A k-means clustering (R package stats)^147^ grouped the variables by importance; the number of clusters (n = 4) was chosen to maximize homogeneity within a cluster and minimize homogeneity between clusters (Figure 4B). The PCA (R package stats)^147^ summarizes the dataset in new dimensions representing which is the most variable between individuals. RF and PCA were run on the same datasets. As both methods cannot handle missing data; they were run on a selection of variables including all animals of the study, and additionally on two other sets with more variables but excluding some groups of animals (see Tables S5, S6, S7 and S8).

One *Tph2*^*+/+*^ animal was excluded from the RGT because it did not sample the options (Table S10). One *Tph2*^*−/−*^ animal was excluded from the odor discrimination test because it did not explore the open field. One group of six *Tph2*^*+/+*^ rats was excluded from RE analysis due to a grid malfunction on days 1 and 2. One group of six *Tph2*^*−/−*^ animals were born from *Tph2*^*−/−*^ dams. In order to control for potential carryover effects of the mother’s genetic background over the offsprings’ behaviors, we compared their results with the results of the other *Tph2*^*−/−*^ animals (born from *Tph2*^*+/−*^ dams). Both groups of *Tph2*^*−/−*^ animals behave similarly in all tests (Wilcoxon rank-sum test, data not shown). They did not form a subgroup different from other *Tph2*^*−/−*^ rats.

## Data Availability

All original data are publicly available in the Zenodo repository: https://doi.org/10.5281/zenodo.4912528. All original codes are publicly available in the Zenodo repository: https://doi.org/10.5281/zenodo.4912528. Any additional information required to reanalyze the data reported in this paper is available from the lead contact Marion Rivalan (marionrivalan@gmail.com) upon request.
