# Supplementary material for: Constitutive depletion of brain serotonin differentially affects rats’ social and cognitive abilities
Source: iScience. 2023 Jan 18;26(2):105998. doi: 10.1016/j.isci.2023.105998 (PMC9926123; doi:10.1016/j.isci.2023.105998)
Supplement: Document S1. Figures S1–S9 and Tables S1–S10 [file mmc1.pdf]

iScience, Volume *26*

Supplemental information

Constitutive depletion of brain serotonin differentially affects rats' social and cognitive abilities

Lucille Alonso, Polina Peeva, Sabrina Stasko, Michael Bader, Natalia Alenina, York Winter, and Marion Rivalan

SUPPLEMENTAL FIGURES

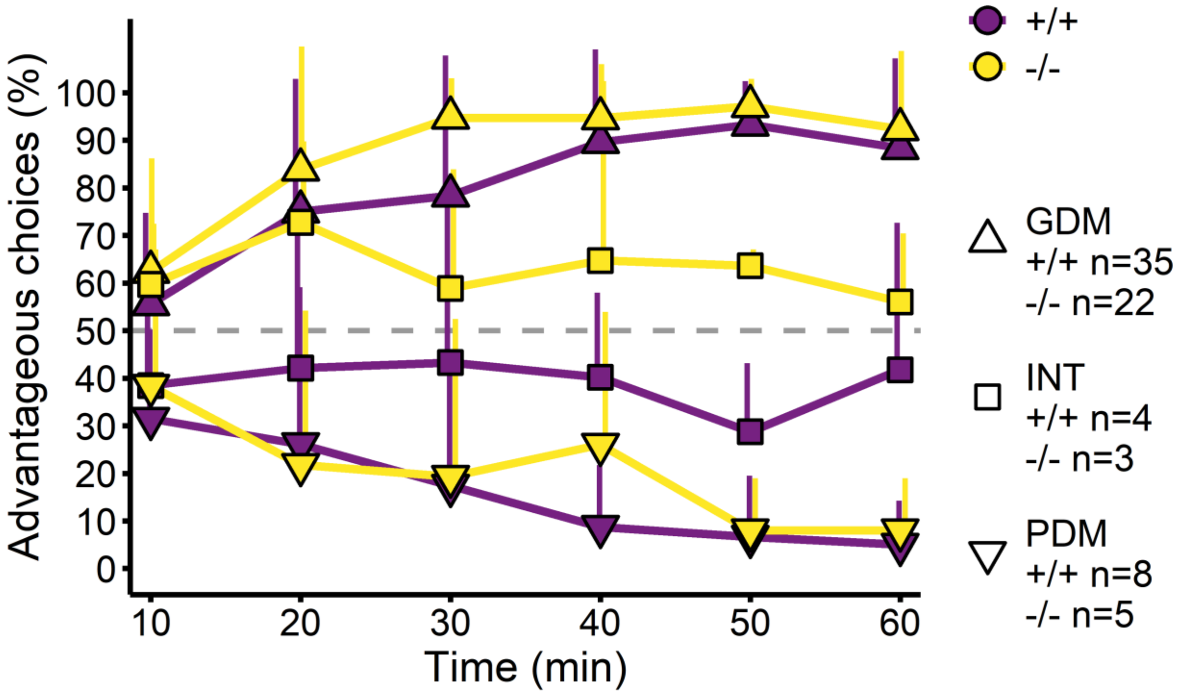

Figure S1. Progression of choices per type of decision maker, related to Figure 1A. Good decision makers (GDM, upward triangle), intermediates (INT, square). and poor decision makers (PDM, downward triangle). Lines indicate mean + SD. *Tph2*$^{+/+}$ in purple and *Tph2*$^{-/-}$ in yellow.

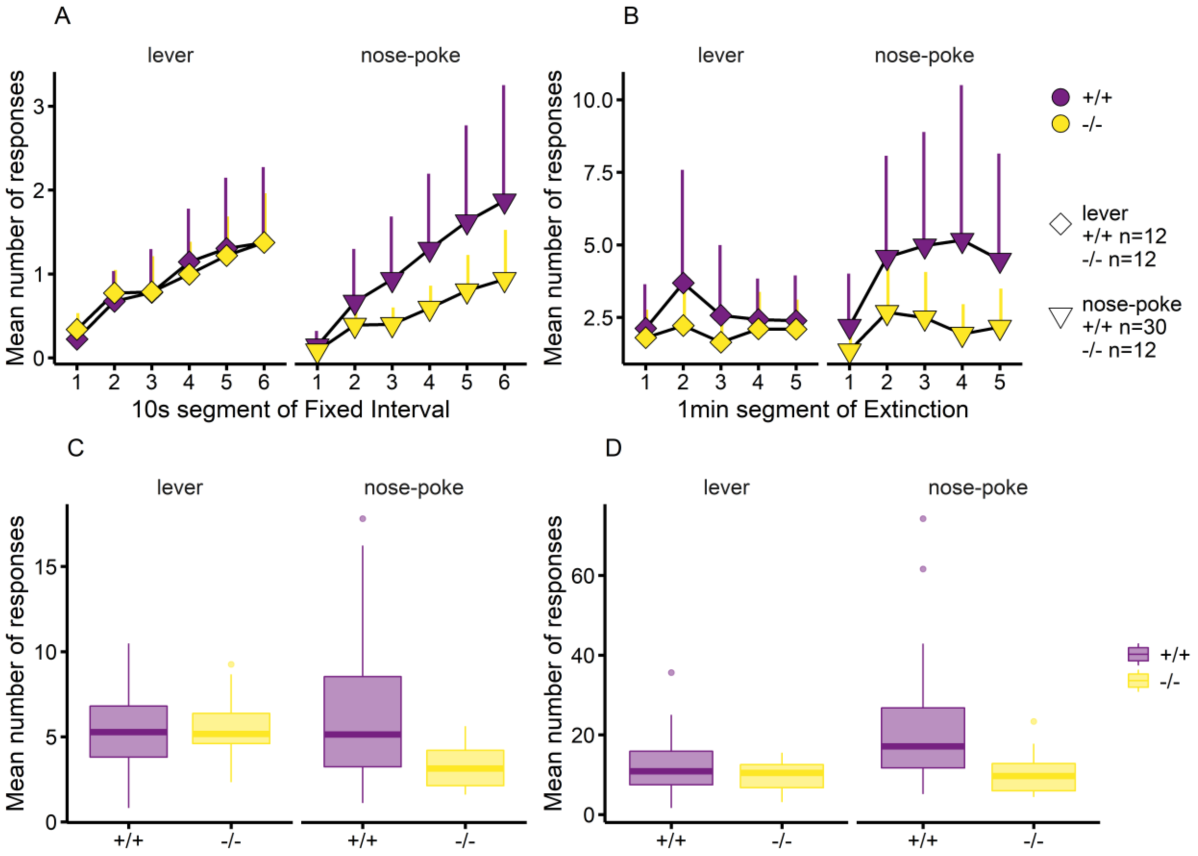

Figure S2. Responses in FIEXT task with lever or nose-poke manipulandum, related to Figure 1. **A-**Mean number of responses during FI (fixed interval) per 10s, lines indicate mean + SD, nose-poke: Wilcoxon rank sum test, W = 269, p-value = 0.012. **B-**Mean number of responses during EXT (extinction) per min, lines indicate mean + SD, nose-poke: Wilcoxon rank sum test, W = 275, p-value = 0.008. **C-**Mean number of responses in FI, *Tph2*$^{-/-}$: lever *vs.* nose-poke: Wilcoxon rank sum test, W = 120, p-value = 0.004. **D-**Mean number of responses in EXT, *Tph2*$^{+/+}$: lever *vs.* nose-poke, Wilcoxon rank sum test, W = 105.5, p-value = 0.039. *Tph2*$^{+/+}$ in purple and *Tph2*$^{-/-}$ in yellow.

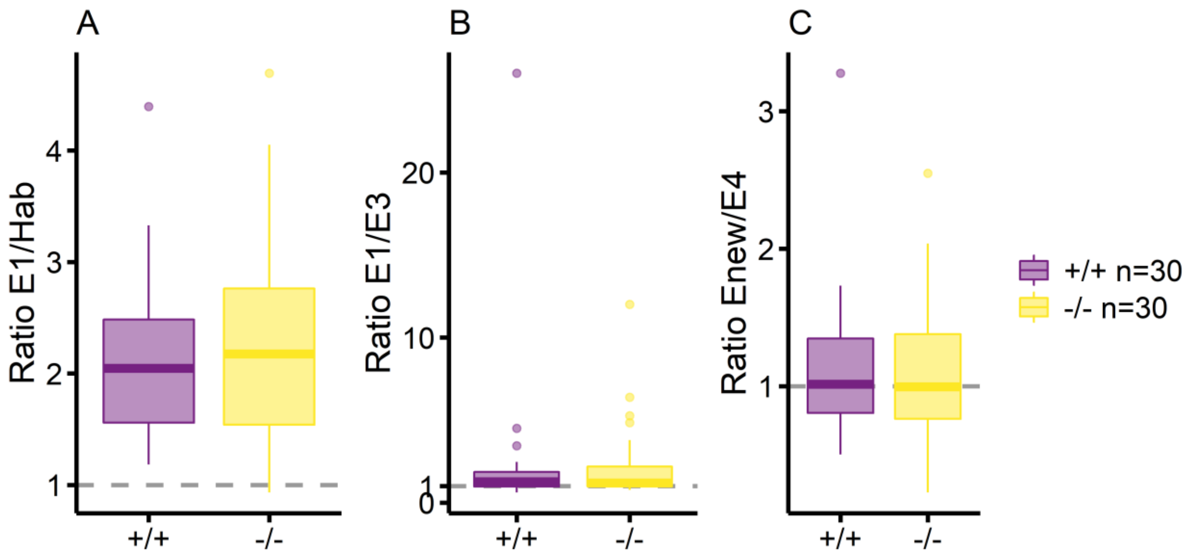

Figure S3. Social recognition task ratios, related to Figure 1G. A-Social preference calculated as the ratio of the interaction time in E1 (first encounter) and hab (habituation) with and without the intruder, Wilcoxon rank sum test with continuity correction, W = 417.5, p-value = 0.6361. **B-**Short term social recognition memory calculated as the ratio of the interaction time in E1 and in E3 (third encounter) with the same intruder, Wilcoxon rank sum test, W = 423, p-value = 0.6973. **C-**Long term social recognition memory calculated as the ratio of the interaction time on the next day with a new intruder *versus* the familiar intruder as in E1, E2, and E3 in Enew (first encounter with new intruder) and in E4 (fourth encounter with familiar encounter), Wilcoxon rank sum test, W = 439, p-value = 0.958. *Tph2*$^{+/+}$ in purple and *Tph2*$^{-/-}$ in yellow.

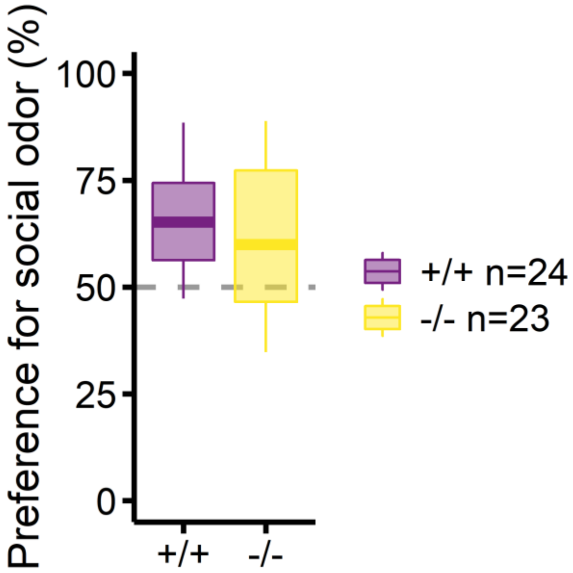

Figure S4. Preference for the social odor in the odor discrimination test, related to Figure 1G. Wilcoxon sign test, +/+: 95CI [57, 71], p-value < 0.001; -/-: 95CI [50, 76], p-value = 0.034 and Wilcoxon rank sum test with continuity correction, W = 330, p-value = 0.3921. *Tph2*$^{+/+}$ in purple and *Tph2*$^{-/-}$ in yellow.

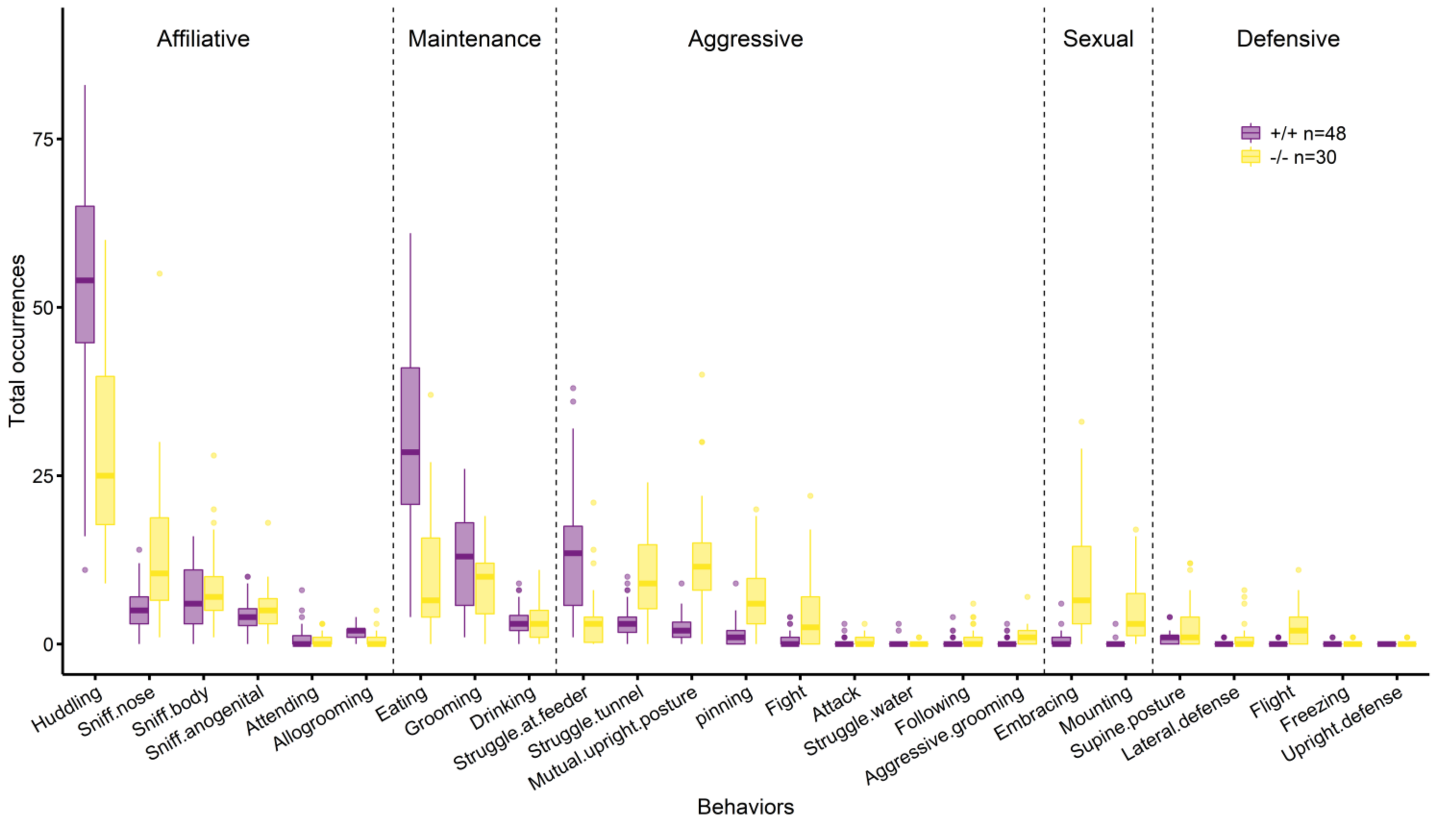

Figure S5. All behaviors expressed in the VBS, related to Figure 3A. Vertical dash lines separate the categories of behaviors. Concerning the affiliative behaviors: huddling: Wilcoxon rank sum test with continuity correction, W = 1240.5, p-value = 9.172e-08; allogrooming: Wilcoxon rank sum test with continuity correction, W = 1086, p-value = 9.623e-05; sniffing directed to the nose: Wilcoxon rank sum test with continuity correction, W = 315.5, p-value = 3.177e-05. Concerning the maintenance behaviors, eating: Wilcoxon rank sum test with continuity correction, W = 1267, p-value = 1.962e-08; grooming: Wilcoxon rank sum test with continuity correction, W = 914.5, p-value = 0.04591, drinking: n.s. Concerning the aggressive behaviors, struggling at feeder: Wilcoxon rank sum test with continuity correction, W = 1227.5, p-value = 1.81e-07; Wilcoxon rank sum test with continuity correction, struggling in tunnel: Wilcoxon rank sum test with continuity correction, W = 233.5, p-value = 5.31e-07, mutual upright posture: Wilcoxon rank sum test with continuity correction, W = 118.5, p-value = 5.426e-10, pinning: Wilcoxon rank sum test with continuity correction, W = 268, p-value = 2.66e-06, fight: Wilcoxon rank sum test with continuity correction, W = 452, p-value = 0.003342, attack: W = 518, p-value = 0.004951, following: Wilcoxon rank sum test with continuity correction, W = 485, p-value = 0.001977, aggressive grooming: Wilcoxon rank sum test with continuity correction, W = 361, p-value = 2.963e-05, struggling at water n.s. Concerning the sexual behaviors, embracing: Wilcoxon rank sum test with continuity correction, W = 88.5, p-value = 1.198e-11; mounting: Wilcoxon rank sum test with continuity correction, W = 113, p-value = 3.823e-13. Concerning the defensive behaviors, overall: Wilcoxon rank sum test with continuity correction, W = 14166, p-value = 2.977e-06. *Tph2*$^{+/+}$ in purple and *Tph2*$^{-/-}$ in yellow.

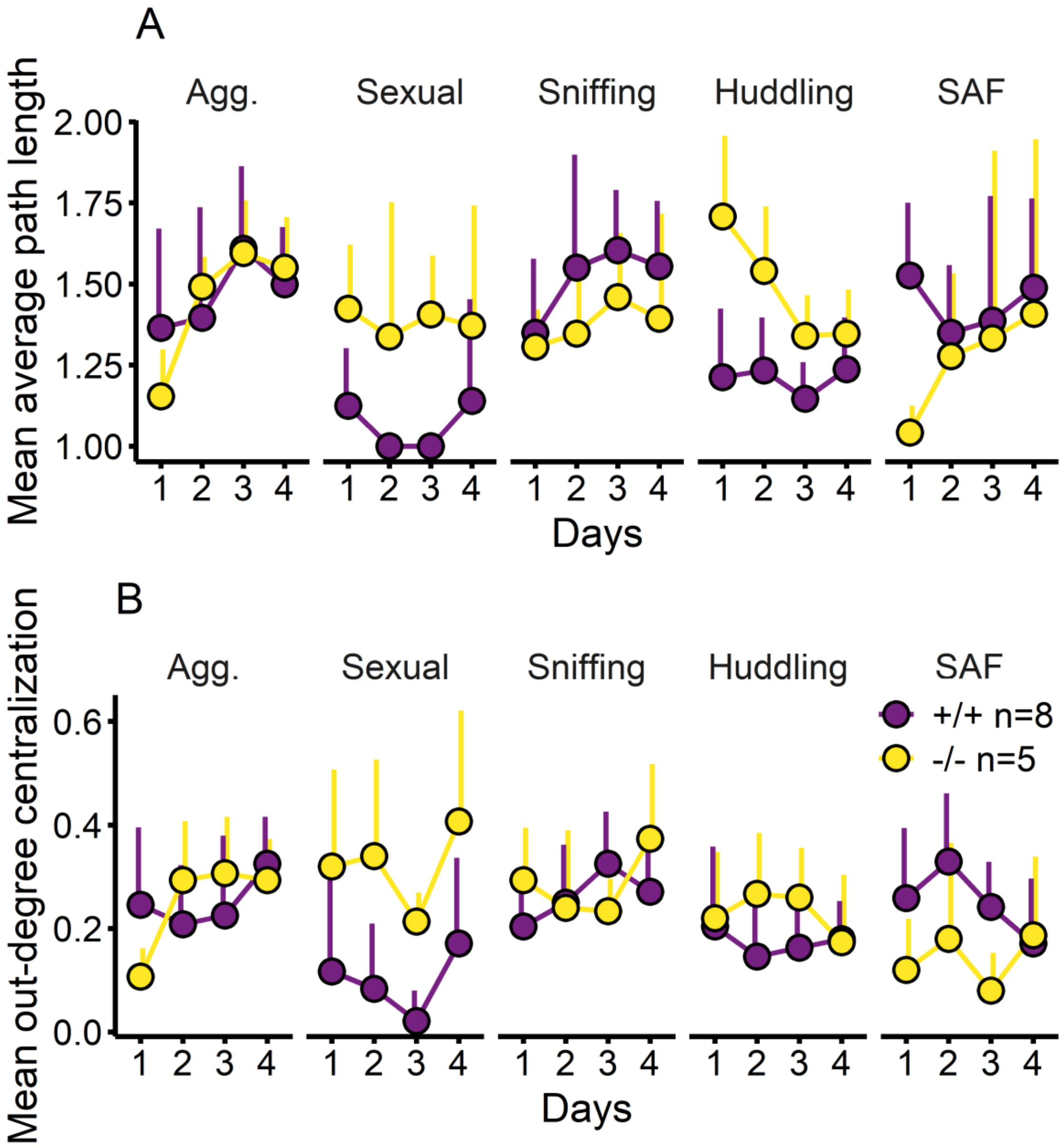

Figure S6. Average path length and out-degree centralization, related to Figure 3B. A-Mean average path length for each group of behavior. Huddling: lmer *day*: F(3,14) = 4, p-value = 0.0208; Agg: lmer *day*: F(3,10) = 13, p-value < 0.001; Sniffing: lmer *genotype*: F(1,43) = 5, p-value = 0.0319. **B-**Mean out-degree centralization for each group of behavior. SAF: lmer *genotype*: F(1,44) = 8, p-value = 0.0072; Sexual: *lmer genotype*: F(1,41) = 29, p-value < 0.001. "Agg." for general aggression, "SAF" for struggling at the feeder. The global parameters are calculated for the VBS groups. Lines indicate mean + SD. *Tph2*$^{+/+}$ in purple (n = 8 groups) and *Tph2*$^{-/-}$ in yellow (n = 5 groups).

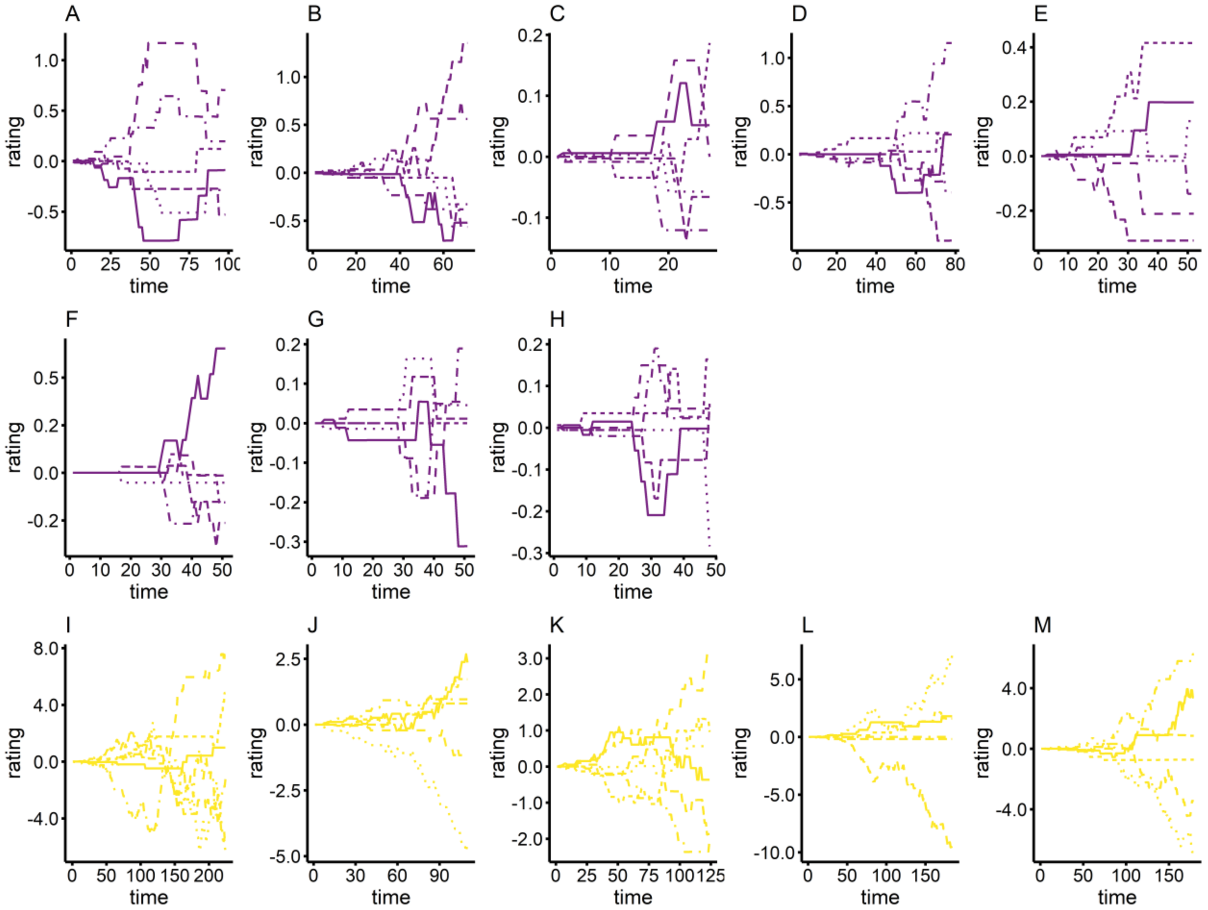

Figure S7. Glicko rating for each VBS group, related to Figure 3D. *Tph2*$^{+/+}$ in purple (panels A-H) and *Tph2*$^{-/-}$ in yellow (panels I-M). One group had two dominant animals (with a rating higher than 1/3 of the maximum rating contrast of the group after 4 days of VBS experiment, panel I).

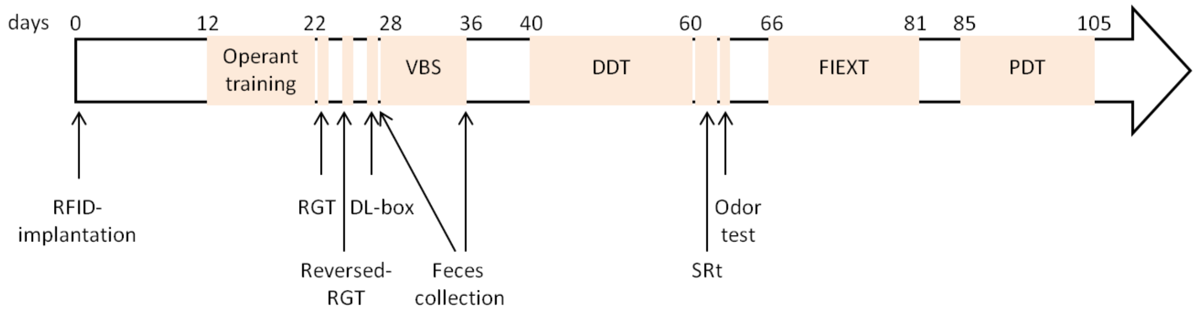

Figure S8. Order of tests, related to METHOD DETAILS. Radio-frequency identification (RFID), rat gambling task (RGT), dark-light box test (DL-box), automated visible burrow system (VBS), delay discounting task (DDT), social recognition test (SRt), odor discrimination test (odor test), fixed-interval and extinction schedule of reinforcement test (FIEXT), probability discounting task (PDT).

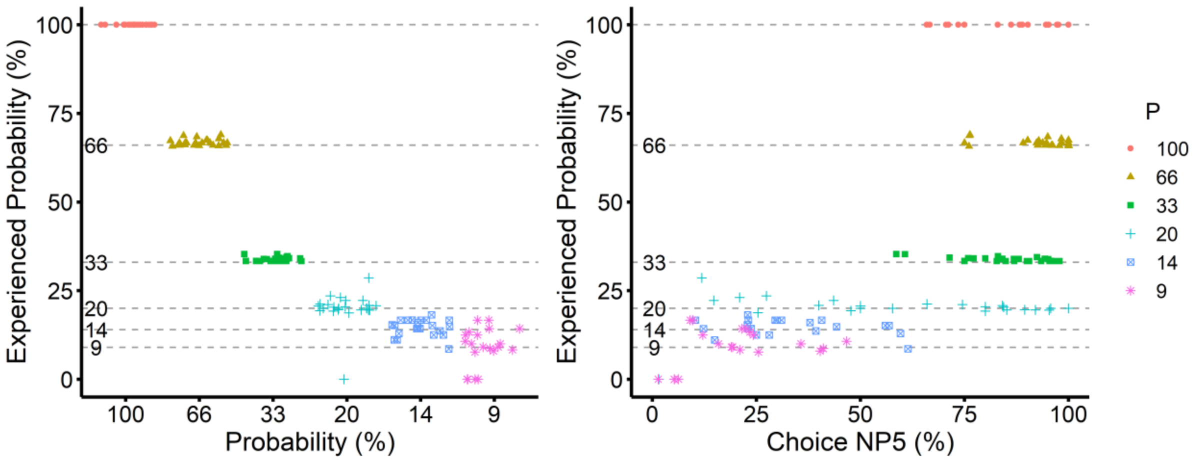

Figure S9. Variation in experienced probability in the probability discounting task, related to Figure 1F. Left panel-Experienced probability at each theoretical probability. **Right panel**-Experienced probability depending on the preference for NP5 (large and uncertain reward). "P" for probability. Data from batch 12, n = 12 including n = 6 +/+ and n = 6 −/−.

SUPPLEMENTAL TABLES

Table S1. One sample t-test results, related to Figure 1A. Mean preference is compared to 50%.

**Genotype**	**Time point**	**0.95 CI**	**p-value**
+/+	10	[43.8, 56.2]	0.9965
−/−	10	[48.8, 67.3]	0.0853
−/−	20	[53.7, 73.9]	**0.008**
−/−	20	[59.5, 85.2]	**< 0.001**
+/+	30	[53.6, 75.8]	**0.010**
−/−	30	[66.2, 90.9]	**< 0.001**
+/+	40	[60.8, 82.4]	**< 0.001**
−/−	40	[68.4, 91.6]	**< 0.001**
+/+	50	[62.2, 83.8]	**< 0.001**
−/−	50	[66.1, 91.8]	**< 0.001**
+/+	60	[59.2, 81.1]	**< 0.001**
−/−	60	[60.4, 87.7]	**0.001**

Table S2. Comparison of GDM and PDM in all tests, related to Figure 1, Figure 2 and Figure 3. All effect sizes (cohen's d) of GDM vs PDM in *Tph2*$^{+/+}$ and *Tph2*$^{–/–}$ for all other tests than RGT and reversed-RGT. Nota bene: the number of PDM individuals varied from n = 8 to n = 1 in *Tph2*$^{+/+}$ group (only n = 3 in the PDT, odor discrimination test and Dark-Light box. and n = 5 in the FIEXT with nose-poke hole and n = 1 in the FIEXT with lever). The number of PDM individuals varied from n = 5 to n = 4 in *Tph2*$^{–/–}$ group (one PDM individual excluded in the odor discrimination test).

**Trait**	**Test**	**Parameter**	***Tph2*$^{+/+}$ GDM vs. PDM cohen's d**	***Tph2*$^{–/–}$ GDM vs. PDM cohen's d**
Impulsive decision making	DDT	AUC DDT	-0.29 (small)	-0.34 (small)
Risky decision making	PDT	AUC PDT	-0.36 (small)	-0.07 (negligible)
Anticipator activity	FI	Mean number of responses	-0.11 (negligible)	-0.52 (medium)
Perseverative activity	EXT	Mean number of responses	-0.34 (small)	-0.23 (small)
Social preference	SRt	Ratio interaction times E1/Hab	0.64 (medium)	0.16 (negligible)
Social short term recognition	SRt	Ratio interaction times E1/E3	0.33 (small)	0.58 (medium)
Social long term recognition	SRt	Ratio interaction times Enew/E4	0.27 (small)	0.07 (negligible)
Exploration Dark-Light box	DL box	Time spent in light compartment	-0.34 (small)	-0.39 (small)
Risk taking Dark-Light box	DL box	Risk taking index	0.70 (medium)	0.62 (medium)
Activity	VBS	Total distance	0.01 (negligible)	-0.09 (negligible)
Entropy	VBS	Total roaming entropy	0.03 (negligible)	-0.25 (small)
Weight loss	VBS	Percentage of weight loss	-0.62 (medium)	-0.17(negligible)
Corticosterone response	VBS	Percentage of corticosterone after the stay in the VBS	0.29 (small)	0.27 (small)
Huddling behavior	VBS	Occurrences huddling behavior	0.77 (medium)	-0.22(small)
Sniffing behavior	VBS	Occurrences sniffing behavior	-0.20 (small)	0.50 (small)
Eating behavior	VBS	Occurrences eating behavior	-0.16 (negligible)	-0.69 (medium)
Grooming behavior	VBS	Occurrences grooming behavior	0.04 (negligible)	0.19 (negligible)
Struggle At Feeder behavior	VBS	Occurrences SAF behavior	0.11 (negligible)	0 (negligible)
General aggression behaviors	VBS	Occurrences gen. aggression behaviors	-0.14(negligible)	-0.43 (small)
Sexual behaviors	VBS	Occurrences sexual behaviors	**-0.80 (large)**	0.35 (small)
Influence over Huddling network	VBS	HUB centrality huddling behavior	**0.86 (large)**	0.18 (negligible)
Influence over Sniffing network	VBS	HUB centrality sniffing behavior	-0.44 (small)	**1.13 (large)**
Influence over SAF network	VBS	HUB centrality SAF behavior	-0.01 (negligible)	-0.70 (medium)
Influence over gen. aggression network	VBS	HUB centrality gen. aggression behaviors	-0.15 (negligible)	-0.19 (negligible)
Influence over Sexual network	VBS	HUB centrality sexual behaviors	-0.52 (medium)	-0.02 (negligible)

Table S3. Lmer results, related to Figure 3B. Effect of genotype, day and interaction on network density and effect of day on network density for each genotype tested with lmer for Figure 3B.

**Network**	**effect**	**F value (degree of freedom)**	**p-value**
General aggression	genotype	F(1, 43) = 40.9	**< 0.001**
General aggression	day	F(3, 38) = 6.2	**0.0015**
General aggression	genotype x day	F(3, 38) = 7.2	**< 0.001**
General aggression	day (+/+ only)	F(3, 23) = 1.9	0.165
General aggression	day (−/− only)	F(3, 12) = 12.6	**< 0.001**
Sexual behaviors	genotype	F(1, 44) = 167	**< 0.001**
Sexual behaviors	day	F(3, 38) = 13.7	**< 0.001**
Sexual behaviors	genotype x day	F(3, 38) = 11	**< 0.001**
Sexual behaviors	day (+/+ only)	F(3, 23) = 2	0.1439
Sexual behaviors	day (−/− only)	F(3, 13) = 13	**< 0.001**
Sniffing	genotype	F(1, 32) = 15	**< 0.001**
Sniffing	day	F(3, 22) = 4.7	**0.0098**
Sniffing	genotype x day	F(3, 22) = 1.3	0.2943
Sniffing	day (+/+ only)	F(3, 20) = 9	**< 0.001**
Sniffing	day (−/− only)	F(3, 11) = 4	**0.0365**
Huddling	genotype	F(1, 43) = 32.5	**< 0.001**
Huddling	day	F(3, 38) = 5.9	**0.0019**
Huddling	genotype x day	F(3, 38) = 4.7	**0.0064**
Huddling	day (+/+ only)	F(3, 23) = 0.2	0.8919
Huddling	day (−/− only)	F(3, 12) = 8.5	**0.0027**
Struggling at feeder	genotype	F(1, 43) = 15.2	**< 0.001**
Struggling at feeder	day	F(3, 38) = 1.2	0.2969
Struggling at feeder	genotype x day	F(3, 38) = 0.8	0.4805
Struggling at feeder	day (+/+ only)	F(3, 23) = 2	0.1383
Struggling at feeder	day (−/− only)	F(3, 14) = 0.2	0.8916

Table S4. Random forests on the three datasets, related to Figure 4B. Parameters of the Random forest for the three versions of the datasets. Version 1 corresponds to Fig. 4B.

**Versions of the dataset**	**Number of *Tph2*$^{+/+}$**	**Number of *Tph2*$^{-/-}$**	**Number of variables**	**Explanation**	**Mean accuracy**	**SD**
1	n = 48	n = 30	17	All individuals, omitting PDT, SRt, DL-box and FI-EXT	98.53 %	0.54
2	n = 23	n = 24	24	omitting FI-EXT	100 %	0
3	n = 17	n = 18	26	All variables	100 %	0

Table S5. Principal Component Analysis on the three datasets, related to Figure 4A. Parameters of the Principal Component Analysis for the three versions of the datasets. PC for principal component (or dimension). Version 1 corresponds to Fig. 4A.

**Versions of the dataset**	**Number of *Tph2*$^{+/+}$**	**Number of *Tph2*$^{-/-}$**	**Number of variables**	**Explanation**	**Variance for PC1**	**Number of PC to reach 80%**
1	n = 48	n = 30	17	All individuals, omitting PDT, SRt, DL-box and FIEXT	23.13 %	8 PC
2	n = 23	n = 24	24	omitting FIEXT	21.28 %	10 PC
3	n = 17	n = 18	26	All variables	18.71 %	10 PC

Table S6. Gini indexes of the Random forests, related to Figure 4B. Order of variables per importance (Mean Gini index) for the three versions of the datasets. Version 1 corresponds to Fig. 4B. Most important variables in bold. Variables from classical tests (not VBS) in grey. Total occurrences of sexual behaviors (Sexual), percentage of weight variation (Weight), percentage of corticosterone metabolites variation (Corticosterone), total distance traveled (Distance), total occurrences of defensive behaviors (Defensive), total roaming entropy (Entropy), total occurrences of maintenance behaviors (Maintenance), total occurrences of aggressive behaviors (Aggressive), total preference for open area (Pref.open area), (Affiliative) total occurrences of affiliative behaviors, (AUC.DDT) area under the curve in the DDT, Hub centrality in aggression network (HUB.agg.), flexibility score in reversed-RGT (Flexibility), preference in last 20 min of RGT (RGT), latency to collect pellet in RGT (Latency RGT), Blanchard dominance score (Blanchard), time spent in the light compartment of the DL-box (timeL.DL), index of risk taking in the DL-box test (Risk.taking.DL), area under the curve in the PDT (AUC.PDT), social preference ratio (Socpref), social preference ratio on day 2 of SRt (Socpref.day2), short-term social recognition memory (STM), long-term social recognition memory (LTM), total number of responses in the fixed-interval of FIEXT (FI), total number of responses in the extinction of FIEXT (EXT).

**Version 1**			**Version 2**			**Version 3**		
	Variable	Mean Gini		Variable	Mean Gini		Variable	Mean Gini
1	**Sexual**	**7.8004641**	1	**Corticosterone**	**3.82187434**	1	**Corticosterone**	**3.61567559**
2	**Weight**	**7.7982767**	2	Glicko rating	3.56926427	2	**Affiliative**	**2.97762276**
3	Corticosterone	3.9158579	3	Affiliative	3.44096951	3	Glicko rating	2.14805505
4	Distance	3.3537214	4	Blanchard	2.41522225	4	Pref.open area	2.04673183
5	Entropy	2.8193245	5	Pref.open area	1.8968134	5	Blanchard	1.37556659
6	Defensive	2.6518695	6	Sexual	1.72011537	6	Sexual	0.76658915
7	Maintenance	2.0825444	7	Defensive	0.9881357	7	Defensive	0.60835491
8	Glicko rating	1.4775315	8	Distance	0.96160999	8	Entropy	0.51658714
9	Aggressive	1.242993	9	Entropy	0.90738647	9	HUB.agg.	0.47006703
10	Pref.open area	1.0495323	10	HUB.agg.	0.76355717	10	Distance	0.31380522
11	Affiliative	0.879558	11	timeL.DL	0.31995666	11	Maintenance	0.2979113
12	AUC.DDT	0.3668323	12	Socpref	0.26738162	12	AUC.PDT	0.26733996
13	HUB.agg.	0.2483838	13	Risk.taking.DL	0.24820501	13	timeL.DL	0.1686874
14	Flexibility	0.2315216	14	Weight	0.24772993	14	EXT	0.1530195
15	RGT	0.2251097	15	Maintenance	0.24716998	15	Latency RGT	0.14738444
16	Latency RGT	0.1886158	16	AUC.PDT	0.20668295	16	FI	0.14641028
17	Blanchard	0.1310371	17	LTM	0.16000447	17	Risk.taking.DL	0.14597387
			18	Latency RGT	0.15219654	18	RGT	0.12270368
			19	Flexibility	0.12945049	19	Socpref.day2	0.09864794
			20	Socpref.day2	0.12204077	20	Aggressive	0.09359775
			21	AUC.DDT	0.1074535	21	Weight	0.09181905
			22	STM	0.10264048	22	LTM	0.09012947
			23	Aggressive	0.09920503	23	Flexibility	0.08875745
			24	RGT	0.09322517	24	STM	0.08836513
						25	AUC.DDT	0.07651249
						26	Socpref	0.06485988

Table S7. Contribution of the variables to PC1, related to Figure 4A. Order of variables per contribution to Dimension 1 (PC1) of the Principal Component Analysis (rotation) for the three versions of the datasets. Version 1 corresponds to Fig. 4A. Most important variables in bold. Variables from classical tests in grey. See abbreviations in the text of Table S6.

Version 1			Version 2			Version 3		
	Variables	Rotation PC1		Variables	Rotation PC1		Variables	Rotation PC1
1	**Weight**	**-0.436**	1	**Sexual**	**-0.351**	1	**Corticosterone**	**0.358**
2	**Maintenance**	**0.362**	2	**Affiliative**	**-0.344**	2	**Pref.open area**	**0.349**
3	**Entropy**	**0.353**	3	**Corticosterone**	**-0.318**	3	**Affiliative**	**0.336**
4	**Corticosterone**	**-0.348**	4	**timeL.DL**	**-0.308**	4	**Sexual**	**0.315**
5	**Defensive**	**-0.327**	5	Distance	-0.290	5	Defensive	-0.257
6	**Sexual**	**-0.325**	6	Socpref	-0.283	6	Glicko rating	-0.254
7	Distance	-0.259	7	Blanchard	-0.265	7	timeL.DL	0.254
8	Aggressive	-0.251	8	Pref.open area	0.264	8	Distance	0.254
9	Pref.open area	0.146	9	Risk.taking.DL	0.237	9	FI	0.245
10	Flexibility	-0.127	10	Defensive	0.228	10	Blanchard	0.242
11	Blanchard	0.124	11	Glicko rating	0.211	11	Risk.taking.DL	-0.218
12	RGT	-0.113	12	Weight	-0.194	12	AUC.PDT	-0.184
13	Affiliative	0.105	13	LTM	-0.157	13	EXT	-0.138
14	AUC.DDT	0.089	14	STM	-0.109	14	Socpref	-0.119
15	HUB.agg.	-0.054	15	AUC.PDT	0.089	15	LTM	0.105
16	Latency RGT	-0.052	16	Latency RGT	0.082	16	Entropy	-0.096
17	Glicko rating	-0.021	17	Entropy	0.082	17	Weight	0.094
			18	Aggressive	0.077	18	RGT	-0.080
			19	Socpref.day2	-0.051	19	Latency RGT	-0.068
			20	HUB.agg.	-0.025	20	Flexibility	0.045
			21	AUC.DDT	0.012	21	HUB.agg.	0.041
			22	Maintenance	-0.005	22	Socpref.day2	0.030
			23	Flexibility	0.003	23	Aggressive	0.027
			24	RGT	0.003	24	AUC.DDT	-0.014
						25	STM	-0.010
						26	Maintenance	-0.005

Table S8. Comparison between VBS impairments and descriptions of human symptoms of mental disorders, related to Figure 4. Impulse control disorders (ICDs) are associated with anxiety disorders, obsessive-compulsive disorder (OCD), depression, ADHD, Tourette syndrome and Parkinson's disease.[1–6] Stress and anxiety disorders share a high level of comorbidity with ICDs. Reduced territory is only one aspect of the hypervigilant defensive profile of *Tph2*$^{-/-}$ rats. We included the full hypervigilant defensive profile to show the parallel with the human aspects of hypervigilance and defensive behaviors.

Variable (VBS parameter)	Corresponding human symptom	Disease
**Sexual**<br>(total occurrences of sexual behaviors)	Uncontrollable repetitive sexual behavior, repetitive aggression	ICD[7,8]
**Maintenance**<br>(total occurrences of maintenance behaviors: eating, drinking, grooming)	Neglect of personal care<br>(due to repetitive behavior)	ICD[7,8]
**Weight**<br>(percentage of weight variation)	Neglect of health and personal care<br>(due to repetitive behavior)	ICD[7,8]
**Corticosterone**<br>(percentage of corticosterone metabolites variation)	Cortisol disturbances,<br>stress triggering impulses	ICD, Alcohol use disorder, Borderline personality disorder[3,9–13]
**Entropy**<br>(total roaming entropy)<br><br>**Hypervigilant defensive profile**:<br>• smaller territory (roaming entropy) excluding food zones and favoring hiding places and escape routes (place preference),<br>• higher activity (distance),<br>• higher sniffing behavior,<br>• inhibition of maintenance behaviors and of huddling,<br>• increased stress (corticosterone)	Hypervigilance,<br>attentional bias of vigilance (faster or persistent focus of attention on threatening stimuli),<br>repetitive checking behavior,<br>generalization of fear responses (over-reaction to harmless stimuli disturbing daily life, avoidance of situations with negative expectation)	ICD,[14]<br>OCD,<br>Post-traumatic stress disorder (PTSD),<br>Generalized anxiety disorder[1,4,15,16]
**Defensive**<br>(total occurrences of defensive behaviors)	Hypervigilance,<br>attentional bias of vigilance (faster or persistent focus of attention on threatening stimuli),<br>repetitive checking behavior,<br>generalization of fear responses (over-reaction to harmless stimuli disturbing daily life, avoidance of situations with negative expectation)	ICD,[14]<br>OCD,<br>Post-traumatic stress disorder (PTSD),<br>Generalized anxiety disorder[1,4,15,16]

Table S9: ARRIVE checklist, related to METHOD DETAILS following the guidelines 2.0 [17]

Item	Recommendation	Section
Study design 1<br>For each experiment, provide brief details of study design including:	a. The groups being compared, including control groups. If no control group has been used, the rationale should be stated.	Experimental model, Table S10
	b. The experimental unit (e.g. a single animal, litter, or cage of animals).	Introduction §3
Sample size 2	a. Specify the exact number of experimental units allocated to each group, and the total number in each experiment. Also indicate the total number of animals used.	Experimental model, figure captions, Table S10
	b. Explain how the sample size was decided. Provide details of any a priori sample size calculation, if done.	Method details
Inclusion and exclusion criteria 3	a. Describe any criteria used for including and excluding animals (or experimental units) during the experiment, and data points during the analysis. Specify if these criteria were established a priori. If no criteria were set, state this explicitly.	Quantification and statistical analysis, Table S10
	b. For each experimental group, report any animals, experimental units or data points not included in the analysis and explain why. If there were no exclusions, state so.	Quantification and statistical analysis, Table S10
	c. For each analysis, report the exact value of n in each experimental group.	Figure captions, Tables: S2, S4-5, S9
Randomisation 4	a. State whether randomisation was used to allocate experimental units to control and treatment groups. If done, provide the method used to generate the randomisation sequence.	No randomisation see Method details
	b. Describe the strategy used to minimise potential confounders such as the order of treatments and measurements, or animal/cage location. If confounders were not controlled, state this explicitly.	Method details
Blinding 5	Describe who was aware of the group allocation at the different stages of the experiment (during the allocation, the conduct of the experiment, the outcome assessment, and the data analysis).	No blinding see Method details and Quantification and statistical analysis
Outcome measures 6	a. Clearly define all outcome measures assessed (e.g. cell death, molecular markers, or behavioural changes).	Method details
	b. For hypothesis-testing studies, specify the primary outcome measure, i.e. the outcome measure that was used to determine the sample size.	Method details
Statistical methods 7	a. Provide details of the statistical methods used for each analysis, including software used.	Quantification and statistical analysis
	b. Describe any methods used to assess whether the data met the assumptions of the statistical approach, and what was done if the assumptions were not met.	Quantification and statistical analysis
Experimental animals 8	a. Provide species-appropriate details of the animals used, including species, strain and substrain, sex, age or developmental stage, and, if relevant, weight.	Experimental model
	b. Provide further relevant information on the provenance of animals, health/immune status, genetic modification status, genotype, and any previous procedures.	Experimental model
Experimental procedures 9<br>For each experimental group, including controls, describe the procedures in enough detail to allow others to replicate them, including:	a. What was done, how it was done and what was used.	STAR*Methods
	b. When and how often.	STAR*Methods
	c. Where (including detail of any acclimatisation periods).	STAR*Methods
	d. Why (provide rationale for procedures).	Introduction §2-5
Results 10<br>For each experiment	a. Summary/descriptive statistics for each experimental group, with a measure of variability where applicable (e.g.	Results

conducted, including independent replications, report:	mean and SD, or median and range).	
	b. If applicable, the effect size with a confidence interval.	For specific experiments see Table S2

Table S10. Number of individuals in each test, related to METHOD DETAILS. Number of individuals used for each tests and exclusions are described.

**Test**	***Tph2*** $^{+/+}$	***Tph2*** $^{-/-}$	**Comment**
Total number	n = 48	n = 30	8 cohorts and 5 cohorts of 6 animals each.
RGT	n = 47	n = 30	One *Tph2* $^{+/+}$ animal was excluded from the Rat Gambling Task because it did not sample the options.
Reversed-RGT	n = 47	n = 30	One *Tph2* $^{+/+}$ animal was excluded from the Rat Gambling Task because it did not sample the options; its score in the Reversed-RGT was excluded too.
DL-box	n = 24	n = 24	Only 4 *Tph2* $^{+/+}$ and 4 *Tph2* $^{-/-}$ cohorts were used.
Feces collection	n = 48	n = 30	
VBS	n = 48	n = 30	
DDT	n = 48	n = 30	
SRt	n = 30	n = 30	Only 6 *Tph2* $^{+/+}$ and 6 *Tph2* $^{-/-}$ cohorts were used.
Odor test	n = 24	n = 23	Only 4 *Tph2* $^{+/+}$ and 4 *Tph2* $^{-/-}$ cohorts were used.<br><br>One *Tph2* $^{-/-}$ animal did not explore the open field (or the odors), the corresponding missing value was replaced by the median value of the group.
FIEXT	n = 42	n = 24	Only 7 *Tph2* $^{+/+}$ and 4 *Tph2* $^{-/-}$ cohorts were used.
PDT	n = 24	n = 24	Only 4 *Tph2* $^{+/+}$ and 4 *Tph2* $^{-/-}$ cohorts were used.

REFERENCES

1. Weintraub, D., David, A.S., Evans, A.H., Grant, J.E., and Stacy, M. (2015). Clinical spectrum of impulse control disorders in Parkinson's disease. Mov. Disord. *30*, 121–127. https://doi.org/10.1002/mds.26016.

2. Fontenelle, L.F., Mendlowicz, M.V., and Versiani, M. (2005). Impulse control disorders in patients with obsessive-compulsive disorder. Psychiatry Clin. Neurosci. *59*, 30–37. 10.1111/j.1440-1819.2005.01328.x.

3. Seo, D., Lacadie, C.M., and Sinha, R. (2016). Neural Correlates and Connectivity underlying Stress-related Impulse Control Difficulties in Alcoholism. Alcohol. Clin. Exp. Res. *40*, 1884–1894. 10.1111/acer.13166.

4. Dunsmoor, J.E., and Paz, R. (2015). Fear Generalization and Anxiety: Behavioral and Neural Mechanisms. Biol. Psychiatry *78*, 336–343. 10.1016/j.biopsych.2015.04.010.

5. Silva, B., Canas-Simião, H., and Cavanna, A.E. (2020). Neuropsychiatric Aspects of Impulse Control Disorders. Psychiatr. Clin. North Am. *43*, 249–262. 10.1016/j.psc.2020.02.001.

6. Fanning, J.R., Lee, R., and Coccaro, E.F. (2016). Comorbid intermittent explosive disorder and posttraumatic stress disorder: Clinical correlates and relationship to suicidal behavior. Compr. Psychiatry *70*, 125–133. 10.1016/j.comppsych.2016.05.018.

7. World Health Organization (2019). International Classification of Diseases 11th Revision. https://icd.who.int/en.

8. American Psychiatric Association (2013). Diagnostic and Statistical Manual of Mental Disorders, Fifth Edition. Washington DC. https://www.psychiatry.org/psychiatrists/practice/dsm.

9. Grant, J.E., and Potenza, M.N. (2004). Impulse control disorders: clinical characteristics and pharmacological management. Ann. Clin. Psychiatry Off. J. Am. Acad. Clin. Psychiatr. *16*, 27–34. 10.1080/10401230490281366.

10. Buchanan, T.W., McMullin, S.D., Mulhauser, K., Weinstock, J., and Weller, J.A. (2020). Diurnal cortisol and decision making under risk in problem gambling. Psychol. Addict. Behav. J. Soc. Psychol. Addict. Behav. *34*, 218–229. 10.1037/adb0000474.

11. Djamshidian, A., O'Sullivan, S.S., Papadopoulos, A., Bassett, P., Shaw, K., Averbeck, B.B., and Lees, A. (2011). Salivary cortisol levels in Parkinson's disease and its correlation to risk behaviour. J. Neurol. Neurosurg. Psychiatry *82*, 1107–1111. 10.1136/jnnp.2011.245746.

12. Lieb, K., Rexhausen, J., Kahl, K., Schweiger, U., Philipsen, A., Hellhammer, D., and Bohus, M. (2004). Increased diurnal salivary cortisol in women with borderline personality disorder. J. Psychiatr. Res. *38*, 559–565. 10.1016/j.jpsychires.2004.04.002.

13. Cackowski, S., Reitz, A.-C., Ende, G., Kleindienst, N., Bohus, M., Schmahl, C., and Krause-Utz, A. (2014). Impact of stress on different components of impulsivity in borderline personality disorder. Psychol. Med. *44*, 3329–3340. 10.1017/S0033291714000427.

14. Gillig, P.M., and Sanders, R.D. (2011). Higher cortical functions: attention and vigilance. Innov. Clin. Neurosci. *8*, 43–46.

15. Cludius, B., Wenzlaff, F., Briken, P., and Wittekind, C.E. (2019). Attentional biases of vigilance and maintenance in obsessive-compulsive disorder: An eye-tracking study. J. Obsessive-Compuls. Relat. Disord. *20*, 30–38. 10.1016/j.jocrd.2017.12.007.

16. Baldwin, D.V. (2013). Primitive mechanisms of trauma response: an evolutionary perspective on trauma-related disorders. Neurosci. Biobehav. Rev. *37*, 1549–1566. 10.1016/j.neubiorev.2013.06.004.

17. Sert, N.P. du, Ahluwalia, A., Alam, S., Avey, M.T., Baker, M., Browne, W.J., Clark, A., Cuthill, I.C., Dirnagl, U., Emerson, M., et al. (2020). Reporting animal research: Explanation and elaboration for the ARRIVE guidelines 2.0. PLOS Biol. *18*, e3000411. 10.1371/journal.pbio.3000411.
